# Predicting the global risk of chikungunya virus under climate change using ensemble species distribution models

**DOI:** 10.3389/fcimb.2026.1808175

**Published:** 2026-05-27

**Authors:** Qianqian Zhang, Ling Zhang, Yuchang Ma, Ziyi Jiang, Yuhe Si, Tianxing Zhang, Binbin Jin, Fangfang Tao, Yang Wu, Ye Xu

**Affiliations:** 1 The First Affiliated Hospital of Zhejiang Chinese Medical University (Zhejiang Provincial Hospital of Chinese Medicine), Zhejiang Chinese Medical University, Hangzhou, Zhejiang, China; 2 Department of Immunology and Microbiology, Basic Medical College, Zhejiang Chinese Medical University, Hangzhou, Zhejiang, China; 3 Institute of Disinfection and Vector Control, Hangzhou Center for Disease Control and Prevention (Hangzhou Health Supervision Institution), Hangzhou, Zhejiang, China; 4 Guangzhou Customs Technology Center, Guangzhou, China

**Keywords:** *Ae. aegypti*, *Ae. albopictus*, Biomod2, chikungunya virus, climate change, species distribution model

## Abstract

**Introduction:**

Climate change is expanding vector-borne disease ranges, yet Chikungunya virus (CHIKV) risk projections remain limited by single-model uncertainty and lack of vector integration. CHIKV, transmitted by *Ae. aegypti* and *Ae. albopictus*, threatens 1.3 billion people globally, necessitating robust spatiotemporal risk assessment.

**Methods:**

Using hierarchical ensemble modeling in Biomod2, we first projected vector distributions based on 19 bioclimatic variables and elevation, then integrated vector suitability as biological predictors for CHIKV under 16 CMIP6 scenarios (4 SSPs × 4 GCMs, 2021-2100). Eleven algorithms were evaluated and ensembled to minimize uncertainty.

**Results:**

Ensemble models achieved excellent performance (*Ae. aegypti*: AUC = 0.949, TSS = 0.773; *Ae. albopictus*: AUC = 0.934, TSS = 0.764; CHIKV: AUC = 0.909, TSS = 0.659). *Ae. aegypti* distribution was constrained by temperature stability (isothermality, temperature seasonality), while *Ae. albopictus* responded to both temperature and precipitation. CHIKV distribution was primarily vector-driven (84% explanatory power), further modulated mainly by the mean temperature of wettest quarter. Currently, 21.26% of global land area (139 countries) faces CHIKV risk, concentrated in tropical/subtropical zones. Future projections reveal northward expansion into temperate regions (northeastern North America, central Europe, East Asia), but extreme warming (SSP585) may contract tropical habitats via thermal stress.

**Discussion:**

Multi-model projections identify region-specific invasion risks, with previously unaffected temperate areas emerging as high-priority surveillance zones by 2100. These findings provide actionable risk maps for targeted vector control and preparedness strategies in 139 at-risk countries, particularly those lacking population immunity. Model heterogeneity underscores the necessity of ensemble approaches for climate-health policy planning.

## 1 Introduction

Chikungunya virus (CHIKV) is a vector-borne pathogen belonging to the genus *Alphavirus* (family *Togaviridae*), primarily transmitted by *Aedes aegypti* (*Ae. aegypti*) and *Aedes albopictus* (*Ae. albopictus*). The virus derives its name from the Makonde language, describing the clinical characteristic of patients often adopting a crouched posture due to joint pain (Khongwichit et al., 2021). Arthralgia, fever, rash and myalgia are characteristic of the acute phase of infection (Burt et al., 2017). Some patients progress to a chronic phase, presenting with persistent joint pain and other musculoskeletal symptoms (Goupil and Mores, 2016; O'Driscoll et al., 2021).

The geographic extent and outbreak potential of CHIKV are fundamentally determined by its transmission vectors. Historically, CHIKV has primarily relied on *Ae. aegypti* for transmission—a species that prefers human settlements, with strong anthropophilic behavior, serving as a stable vector for sustained transmission in tropical regions (Christofferson et al., 2023). However, global climate change and cross-regional population mobility have greatly influenced the expansion of transmission vectors and the spread of viral variants (Ryan et al., 2019). During the 2005–2006 Indian Ocean outbreak (Charrel et al., 2007), a mutation (E1-A226V) occurred in the E1 protein of the Indian Ocean lineage of the virus (Vazeille et al., 2007). This mutation significantly enhanced the virus’s adaptability to *Ae. albopictus*. Unlike *Ae. aegypti*, *Ae. albopictus* possesses a broader temperature tolerance range and superior field survival capabilities. This enables CHIKV to breach tropical boundaries and spread into temperate regions across Asia, Europe, and the Americas (Mercier et al., 2022; Freppel et al., 2024). Consequently, CHIKV has become a global health threat, with indigenous transmission reported in 114 countries, placing over three-quarters of the world’s population at risk (Bettis et al., 2022). The case fatality rate is approximately 1.3 per thousand (de Souza et al., 2023), resulting in an annual loss of approximately 284,000 disability-adjusted life years (DALYs) (Ribeiro Dos Santos et al., 2025).

This substantial disease burden is projected to escalate under climate change, which is profoundly altering the distribution patterns of infectious diseases (Hoberg and Brooks, 2015). As global surface temperatures rise (Forster et al., 2024), the ecological niches of disease vectors are shifting (Zhang et al., 2024b). Predicting these shifts is critical for proactive public health planning. Against this backdrop, species distribution models (SDMs) have become essential tools for quantifying species-environment relationships and predicting distribution dynamics (Dong et al., 2022; Lawrence et al., 2023). Ensemble models, which integrate multiple independent models, are considered a preferred approach to mitigate algorithmic uncertainty (Araújo and New, 2007; Harris et al., 2024). Biomod2, an R-based analytical platform, integrates several commonly used SDMs, effectively enhancing prediction accuracy and reliability (Thuiller et al., 2009). Despite these technical advancements, the conceptual framework of how variables are structured remains a critical challenge.

Traditional methodologies frequently treat climate, socio-economics, and vectors as parallel predictors in a single-step model (Kang et al., 2025). However, this may overlook the specific cascading effect: climate first shapes vector niches, which subsequently constrain viral transmission potential. Hierarchical modeling frameworks that explicitly integrate vector distributions as biological predictors can better capture these cascading climate-vector-virus dynamics, particularly under non-stationary future climates where vector-climate relationships may shift (Dai et al., 2025).

Using hierarchical ensemble modeling in Biomod2, this study aims to: (1) quantify the relative contributions of climatic and vector factors to CHIKV distribution; (2) project spatiotemporal risk shifts under 16 CMIP6 scenarios (2021-2100), capturing inter-model uncertainty across four shared socioeconomic pathways (SSPs) and four global climate models (GCMs); (3) identify emerging high-risk regions to inform targeted surveillance and vector control strategies. By integrating vector ecology into ensemble projections, we provide mechanistically grounded risk maps for global public health, addressing critical gaps in climate-health adaptation strategies.

## 2 Materials and methods

### 2.1 Occurrence records of CHIKV and vectors

Occurrence records for the vectors (*Ae. aegypti*, *Ae. albopictus*) spanning 2015–2025 were sourced from the Global Biodiversity Information Facility (GBIF)^1^, while CHIKV records spanning 2010–2022 were assembled from HealthMap^2^. We downloaded 15,600 *Ae. aegypti*, 42,170 *Ae. albopictus*, and 13,524 CHIKV occurrence records from these databases worldwide.

Data cleaning involved: (1) removing records lacking geographic coordinates; (2) excluding duplicate records and those located in oceans or outside the species’ known continental ranges. To mitigate spatial sampling bias and reduce spatial autocorrelation, we implemented spatial thinning on the occurrence data using the ‘spThin’ package (v0.2.0) in R. The minimum nearest-neighbor distance of 50 km was applied to the vectors (*Ae. aegypti* and *Ae. albopictus*), effectively breaking spatial autocorrelation and eliminating massive occurrence clusters in heavily sampled regions (Harrington et al., 2005; Vavassori et al., 2019). Conversely, a finer threshold of 10 km was used for CHIKV. Because viral transmission is inherently human-mediated, this finer scale accurately reflects the localized nature of epidemiological outbreaks (Guzzetta et al., 2020). Finally, 1,324, 1,948, and 1,700 *Ae. aegypti*, *Ae. albopictus*, and CHIKV occurrence records were respectively retained for modeling (**Supplementary Figures 1A–C**).

### 2.2 Environmental variables

To analyze and predict the current and future potential distribution areas of CHIKV, we downloaded current climate data (1970–2000) and future climate projections (2021–2040, 2041–2060, 2061–2080, 2081–2100) from WorldClim^3^. The current climate data (10 arcmin spatial resolution) was sourced from WorldClim 2.1, comprising 19 bioclimatic variables (related to temperature and precipitation) and elevation (**Supplementary Table 1**). Despite a temporal mismatch between occurrence records (2010–2025) and the climate baseline (1970–2000), this long-term baseline provides a robust characterization of stable macroecological niches by smoothing interannual climatic variability. Such cross-temporal integration is widely accepted for establishing contemporary species models (Bean et al., 2024; Wang et al., 2025). For future climate data (10 arcmin spatial resolution), we utilized sixteen combinations derived from four shared socioeconomic pathways (SSP126, SSP245, SSP370, and SSP585) and four global climate models (IPSL-CM6A-LR, MIROC6, MRI-ESM2-0, and UKESM1-0-LL). These four SSP pathways span a range of radiative forcing levels, from a low-forcing, sustainable development scenario (SSP126, 2.6 W/m² by 2100) to a high-forcing, fossil-fuel-based development scenario (SSP585, 8.5 W/m² by 2100) (van Vuuren et al., 2011). Similarly, the four GCMs were selected to span a wide range of equilibrium climate sensitivities (ECS): low-to-medium sensitivity models (MIROC6 and MRI-ESM2-0) to high and very high sensitivity models (IPSL-CM6A-LR and UKESM1-0-LL) (Scafetta, 2023). The selection of these 16 SSP-GCM combinations aims to capture the greatest degree of uncertainty in future climate trends, thereby providing a reliable and multi-scenario prediction range for the potential distribution of CHIKV, rather than relying on the prediction results of a single model.

Nineteen bioclimatic variables and elevation were involved in predicting the distribution of the vectors (*Ae. aegypti* and *Ae. albopictus*). Based on these data, distribution of the CHIKV was modeled through the introduction of the predicted vector distributions as biological variables, following a hierarchical modeling approach. In order to reduce multicollinearity, we examined all the variables with the variance inflation factor (VIF) in the R (v4.5.0) software package usdm (v2.1.7). Variables with VIF > 10 were sequentially excluded until all remaining variables had VIF < 10. **Table 1** summarizes the final list of the selected variables and their percent contributions.

**Table 1:** Variable contribution of *Ae. aegypti, Ae. albopictus* and CHIKV.

Variable	Symbol	Percent contribution		
		*Ae. aegypti*	*Ae. albopictus*	CHIKV
Mean diurnal range	bio2	0.77	5.59	0.31
Isothermality	bio3	40.75	28.34	—
Temperature seasonality	bio4	26.75	5.98	1.89
Mean temperature of wettest quarter	bio8	6.35	5.67	9.89
Mean temperature of driest quarter	bio9	6.62	22.08	—
Precipitation of wettest month	bio13	1.98	22.10	0.23
Precipitation of driest Month	bio14	3.51	1.67	0.40
Precipitation seasonality	bio15	1.58	0.44	1.48
Precipitation of warmest quarter	bio18	0.54	0.22	0.16
Precipitation of coldest quarter	bio19	0.55	0.70	0.50
Elevation	Elevation	10.60	7.21	0.75
*Ae. albopictus*	*Ae. albopictus*	—	—	72.47
*Ae. aegypti*	*Ae. aegypti*	—	—	11.92

“—” in the CHIKV column indicates that the variable was excluded from the final model during the stepwise VIF screening process (VIF > 10). “—” in the *Ae. aegypti* and *Ae. albopictus* columns for the bottom two rows indicates that these biological variables were not applicable as predictors for vector species modeling.

### 2.3 Construction and evaluation of species distribution models

We built species distribution models (SDMs) of CHIKV and its vectors with the Biomod2 (version 4.2.6.2) software package in R. To assess model robustness, we constructed three sets of pseudo-absence point collections sampled randomly from the same global terrestrial extent, each equal in number to presence records (Xu et al., 2025a).

We employed 11 algorithms representing diverse modeling paradigms: presence-only methods (MaxEnt, SRE), regression-based approaches (GLM, GAM, MARS), machine learning techniques (RF, GBM, XGBoost, ANN), and classification methods (CTA, FDA). This multi-algorithm framework captures methodological uncertainty and improves ensemble robustness. These algorithms utilized the default parameters of Biomod2. Full parameter settings for all algorithms are provided in **Supplementary Table 2**.

To ensure rigorous model evaluation, we randomly split the occurrence data into a training dataset (75%) and an independent testing dataset (25%). The testing dataset was withheld from the entire modeling process and used only for final model validation. Within the training dataset, we used a repeated random subsample cross-validation strategy to evaluate model performance objectively and fairly. Specifically, in each of the 10 iterations, the training data was randomly split into a calibration set (75%) for model construction and an internal evaluation set (25%) for preliminary assessment (Zhang et al., 2024a; Ma et al., 2025). Accounting for the three pseudo-absence sets described above and 11 modeling algorithms, 330 models were constructed for each species.

**Figure 1:**
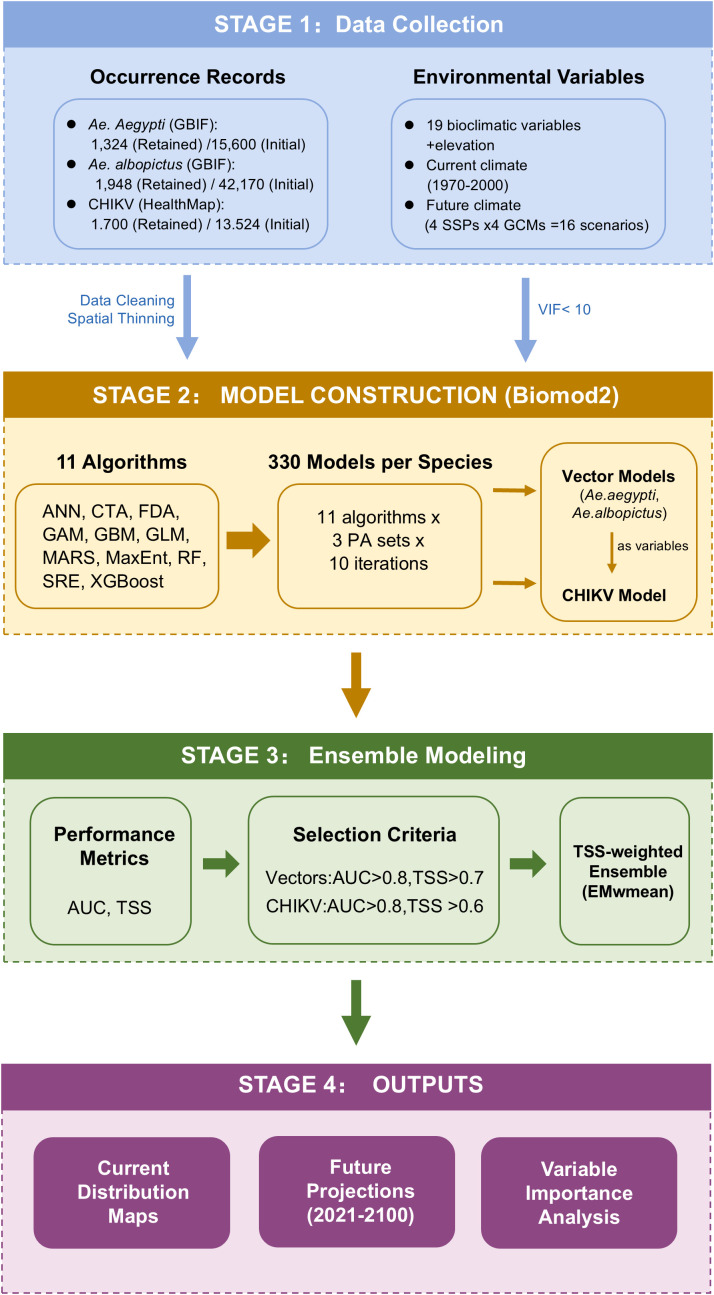
Research framework for modeling the distributions of Chikungunya virus (CHIKV). The process includes: (Stage 1) Data collection of occurrence records and bioclimatic variables; (Stage 2) Model construction using 11 algorithms in the biomod2 package; (Stage 3) Ensemble modeling based on AUC and TSS evaluation; and (Stage 4) Outputs of current distribution, future projections (2021–2100), and variable importance.

The performance of the models was assessed using the area under the receiver operating characteristic curve (AUC) and the true skill statistic (TSS) (Hao et al., 2019). These indicators served as selection criteria for 330 candidate models used to create the TSS-weighted mean ensemble model (EMwmean). Only high-performing models meeting the predefined thresholds were integrated. For the vector models (*Ae. aegypti* and *Ae. albopictus*), models were retained only if they achieved AUC > 0.8 and TSS > 0.7. For the CHIKV model, the selection criteria were relaxed to AUC > 0.8 and TSS > 0.6, accounting for greater spatial uncertainty in disease occurrence data compared to vector records. This combination of strict first-stage thresholds and TSS-weighted ensembling structurally mitigates two-stage error propagation by minimizing baseline errors and penalizing poor algorithms. The complete methodological workflow is illustrated in **Figure 1**.

Furthermore, to objectively validate the necessity and mechanistic superiority of the hierarchical framework, we conducted an ablation study. A baseline, climate-only model for CHIKV was constructed using only the initial bioclimatic variables (excluding the predicted vector suitability layers). The performance metrics (AUC and TSS) and variable contributions of this baseline model were then compared against the hierarchical model.

### 2.4 Habitat suitability classification

We utilized the cutoff value of the ensemble model as the threshold for dividing suitable and unsuitable habitats. The continuous prediction results (representing the probability of species presence, ranging from 0 to 1000) were imported into ArcGIS (v10.8) for further analysis. We employed a percentile-based classification method to divide habitat suitability into four levels (**Supplementary Table 3**): unsuitable habitat (*p<* threshold), low suitability habitat (threshold ≤ *p* < *P_50_*), moderate suitability habitat (*P_50_* ≤ *p* < *P_75_*), and high suitability habitat (*p ≥ P_75_)*. Here, *P_x_* represents the x-th percentile of the environmental suitability values extracted from all presence records in the training dataset (Ong et al., 2021; Wunderlich et al., 2025). This classification scheme was chosen to precisely delineate the ecological risk areas of species distribution and provide a scientific basis for targeted monitoring and early warning systems.

## 3 Results

### 3.1 Evaluation of model performance

**Figure 2:**
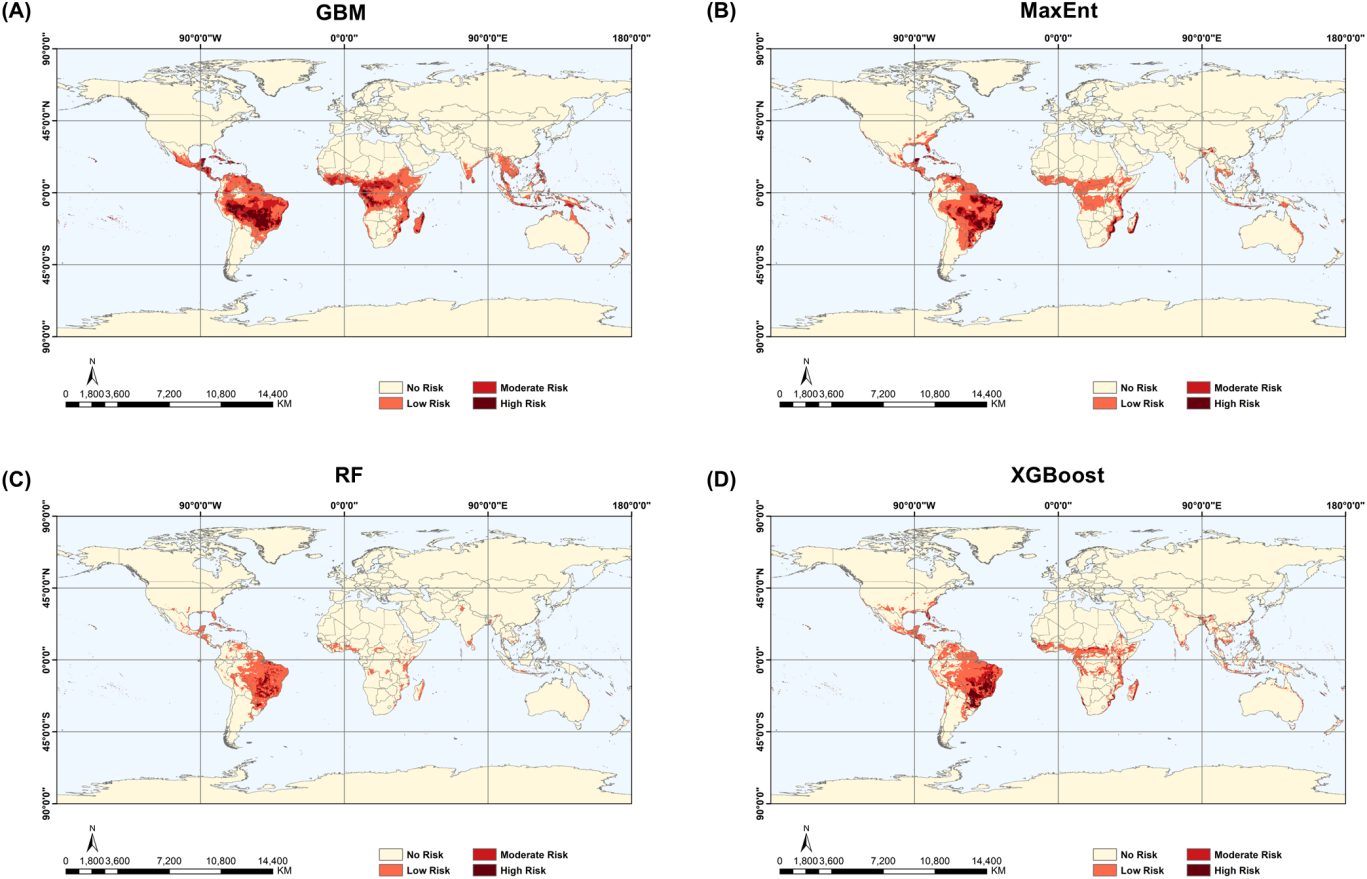
Potential distribution of *Ae. aegypti* predicted by best-performing algorithms. The maps illustrate the habitat suitability for *Ae. aegypti* under current climatic conditions, as projected by four individual algorithms: **(A)** Gradient Boosting Machine (GBM), **(B)** Maximum Entropy (MaxEnt), **(C)** Random Forest (RF), **(D)** Extreme Gradient Boosting (XGBoost).

**Figure 3:**
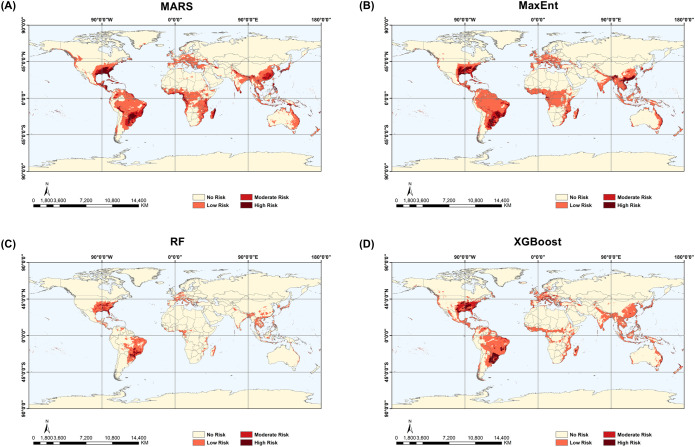
Potential distribution of *Ae. albopictus* predicted by best-performing algorithms. The maps illustrate the habitat suitability for *Ae. albopictus* under current climatic conditions, as projected by four individual algorithms: **(A)** Multivariate Adaptive Regression Splines (MARS), **(B)** Maximum Entropy (MaxEnt), **(C)** Random Forest (RF), **(D)** Extreme Gradient Boosting (XGBoost).

**Figure 4:**
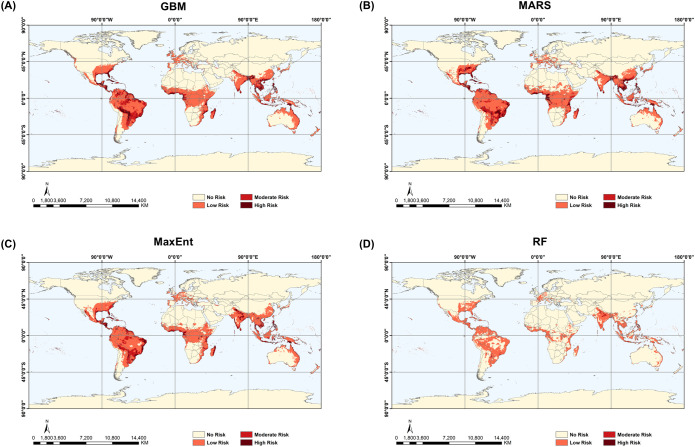
Potential distribution of CHIKV predicted by best-performing algorithms. The maps illustrate the habitat suitability for CHIKV under current climatic conditions, as projected by four individual algorithms: **(A)** Gradient Boosting Machine (GBM), **(B)** Multivariate Adaptive Regression Splines (MARS), **(C)** Maximum Entropy (MaxEnt), and **(D)** Random Forest (RF).

Based on independent test set evaluation, ensemble models achieved excellent discrimination for all three species: *Ae. aegypti* (AUC = 0.949, TSS = 0.773), *Ae. albopictus* (AUC = 0.934, TSS = 0.764), and CHIKV (AUC = 0.909, TSS = 0.659) (**Table 2**). Individual algorithms meeting selection criteria (AUC > 0.8, TSS > 0.7 for vectors; AUC > 0.8, TSS > 0.6 for CHIKV) were integrated into TSS-weighted ensembles, with 6–9 algorithms retained per species (**Table 2**). Across species, RF consistently produced the most conservative predictions, while GBM, MaxEnt, and MARS predicted broader suitable areas (**Figures 2**–**4**; **Supplementary Figures 3**, **5**, **7**). All subsequent analyses used ensemble outputs (EMwmean) to maximize robustness (Araújo and New, 2007; Willcock et al., 2020).

**Table 2:** Model performance metrics of individual and ensemble models.

Algorithms	AUC			TSS		
	*Ae. aegypti*	*Ae. albopictus*	CHIKV	*Ae. aegypti*	*Ae. albopictus*	CHIKV
ANN	0.837 ± 0.037	0.814 ± 0.063	0.786 ± 0.075*	0.670 ± 0.071*	0.611 ± 0.120*	0.563 ± 0.146*
CTA	0.879 ± 0.015	0.849 ± 0.011	0.819 ± 0.019	0.745 ± 0.019	0.674 ± 0.013*	0.616 ± 0.019
FDA	0.896 ± 0.003	0.854 ± 0.002	0.892 ± 0.002	0.716 ± 0.009	0.631 ± 0.006*	0.637 ± 0.002
GAM	0.904 ± 0.002	0.857 ± 0.002	0.893 ± 0.002	0.713 ± 0.013	0.617 ± 0.008*	0.638 ± 0.004
GBM	0.931 ± 0.004	0.910 ± 0.002	0.894 ± 0.003	0.764 ± 0.009	0.711 ± 0.009	0.643 ± 0.006
GLM	0.926 ± 0.002	0.902 ± 0.001	0.894 ± 0.003	0.738 ± 0.006	0.706 ± 0.006	0.651 ± 0.006
MARS	0.931 ± 0.006	0.915 ± 0.003	0.901 ± 0.005	0.756 ± 0.011	0.727 ± 0.008	0.666 ± 0.015
MaxEnt	0.940 ± 0.003	0.916 ± 0.002	0.905 ± 0.002	0.759 ± 0.006	0.720 ± 0.005	0.658 ± 0.006
RF	0.956 ± 0.003	0.945 ± 0.002	0.919 ± 0.003	0.808 ± 0.012	0.778 ± 0.011	0.702 ± 0.010
SRE	0.500 ± 0.000*	0.500 ± 0.000*	0.500 ± 0.000*	0.000 ± 0.000*	0.000 ± 0.000*	0.000 ± 0.000*
XGBoost	0.936 ± 0.006	0.919 ± 0.006	0.892 ± 0.007	0.779 ± 0.014	0.742 ± 0.011	0.665 ± 0.015
EMwmean	0.949	0.934	0.909	0.773	0.764	0.659

Values marked with an asterisk (*) indicate that the algorithm failed to meet the predefined inclusion thresholds (AUC > 0.8 and TSS > 0.7 for vector models; AUC > 0.8 and TSS > 0.6 for the CHIKV model). Algorithms with any metric marked by an asterisk for a given species were explicitly excluded from the final TSS-weighted mean ensemble model (EMwmean).

### 3.2 Contemporary potential distribution of *Ae. aegypti*

Variable importance analysis identified isothermality (bio3, 40.75%), temperature seasonality (bio4, 26.75%), and elevation (10.6%) as the top three predictors for *Ae. aegypti*, collectively accounting for 78.1% of model explanatory power (**Table 1**). Response curves indicated optimal conditions at bio3 (Isothermality, ×100) = 69.12 (suitable range: 64.58–81.84), bio4 (Temperature Seasonality, standard deviation ×100) = 182.88 (suitable range: 45.72–251.46), and elevation = 243.52 m (suitable range: -29 to 843 m) (**Supplementary Figure 2**).

The prediction distributions of the four best-performing algorithms (GBM, MaxEnt, RF, XGBoost) are shown in **Figure 2**, and the prediction results of the other six models are detailed in **Supplementary Figure 3** (SRE was excluded due to insufficient performance). All algorithms identified tropical and subtropical regions as high-suitability areas. However, inter-algorithm variability was evident: compared with RF, the potential suitable areas predicted by GBM and MaxEnt were more extensive. Notably, in Central Africa and Southeast Asia, the suitable areas predicted by these two models were significantly broader than those of RF.

**Figure 5:**
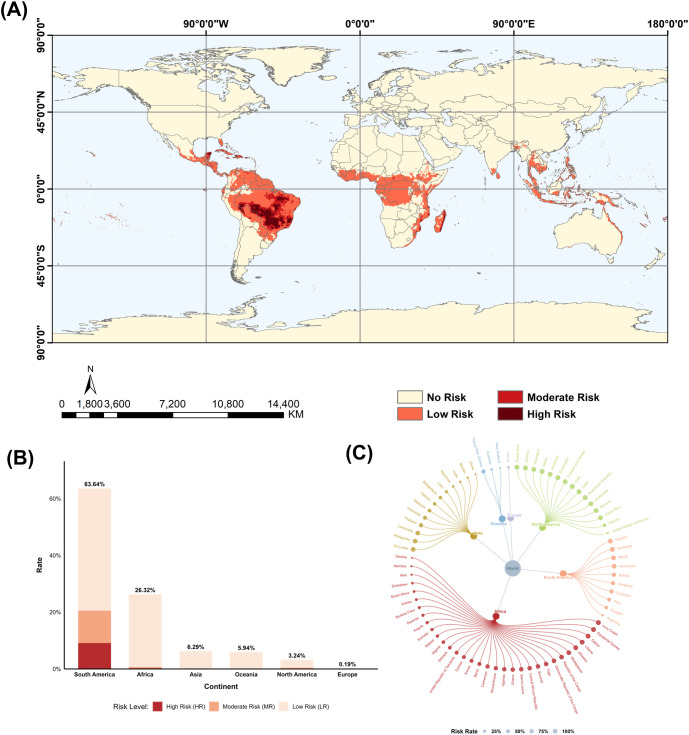
Ensemble model (EMwmean) projections for *Ae. aegypti*. **(A)** Global potential distribution for *Ae. aegypti* under current climatic conditions. **(B)** The predicted risk area percentage of *Ae. aegypti* for each continent. **(C)** Risk rate across affected countries and regions. Circle size represents the level of total risk rates (the sum of low-risk rates, moderate-risk rates and high-risk rates).

The EMwmean prediction (**Figure 5A**) indicated that approximately 9.69% of the global land area is identified as potential habitat for *Ae. aegypti* under current climatic conditions. Habitat suitability was classified into four levels using percentile-based thresholds (**Supplementary Table 3**). High-suitability areas are concentrated in central South America, the Caribbean region of North America, and the southeastern coast of Africa. Moderate and low-suitability areas are distributed in southern North America, northern South America, sub-Saharan Africa, and Southeast Asia.

At the continental scale (**Figure 5B**), South America had the highest proportion of suitable habitat (63.64%), followed by Africa (26.32%), Asia (6.29%), Oceania (5.94%), North America (3.24%), and Europe (0.19%). At the national scale, 78 countries face *Ae. aegypti* risk (**Figure 5C**). The 10 highest-risk countries (ranked by total risk) include Puerto Rico, Jamaica, Nicaragua, Guyana, Belize, Honduras, El Salvador, Suriname, Trinidad and Tobago, and Haiti (complete rankings in **Supplementary Table 4**).

### 3.3 Contemporary potential distribution of *Ae. albopictus*

Variable importance analysis showed that isothermality (bio3, 28.34%), precipitation of wettest month (bio13, 22.10%), and mean temperature of driest quarter (bio9, 22.08%) were the most important predictors for *Ae. albopictus*, collectively accounting for 72.52% of model explanatory power (**Table 1**). The response curve of bio3 showed a hump shape with a suitable range of 29.02–71.79, broader than that of *Ae. aegypti* (64.58–81.84). The response curve of bio9 indicated that -10 °C is a critical threshold; below this temperature, suitability plateaued at a low level. Precipitation of wettest month (bio13) showed that once monthly precipitation exceeds 250 mm, occurrence probability rises sharply and stabilizes at high levels (**Supplementary Figure 4**).

The prediction distributions of the four best-performing algorithms (MARS, MaxEnt, RF, XGBoost) are shown in **Figure 3** and the prediction results of the other six models are detailed in **Supplementary Figure 5** (SRE was excluded). All algorithms showed significant temperate and subtropical distribution patterns. The RF model produced the most conservative predictions, while MARS and MaxEnt predicted broader suitable areas, particularly in northern South America, East Asia, and northeastern Australia.

**Figure 6:**
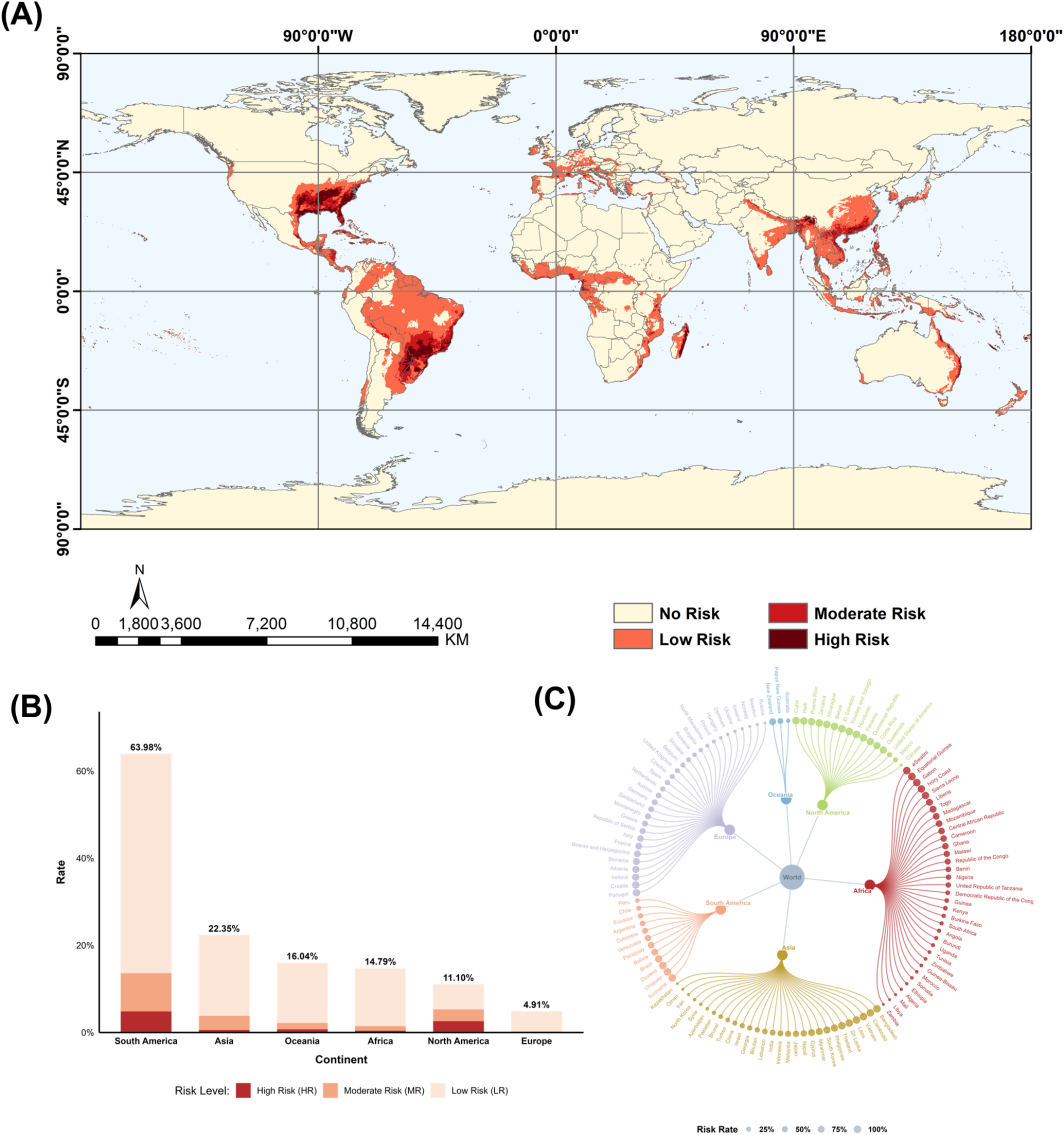
Ensemble model (EMwmean) projections for *Ae. albopictus*. **(A)** Global potential distribution for *Ae. albopictus* under current climatic conditions. **(B)** The predicted risk area percentage of *Ae. albopictus* for each continent. **(C)** Risk rate across affected countries and regions. Circle size represents the level of total risk rates (the sum of low-risk rates, moderate-risk rates and high-risk rates).

The EMwmean prediction (**Figure 6A**) showed that approximately 13.90% of the global land area is identified as potential habitat for *Ae. albopictus*. High-suitability areas are concentrated in the southeastern United States, southeastern South America, and the southeastern coast of Asia. Moderate and low-suitability areas extend inland from high-suitability zones or along coastal frontiers, including the Mediterranean coast of Europe, the Gulf of Guinea, southeastern Africa, and eastern Oceania.

At the continental scale (**Figure 6B**), South America had the highest proportion (63.98%), followed by Asia (22.35%), Oceania (16.04%), Africa (14.79%), North America (11.10%), and Europe (4.91%). At the national scale, *Ae. albopictus* is potentially present in 126 countries and regions (**Figure 6C**). Notable high-risk countries include Equatorial Guinea, Madagascar, Gabon, Eswatini, and Ivory Coast in Africa; Vietnam, Bhutan, Myanmar, Georgia, and China in Asia; France, Italy, and Portugal in Europe (complete rankings in **Supplementary Table 5**).

### 3.4 Contemporary potential distribution of CHIKV

The global distribution of CHIKV is primarily driven by vector suitability, which collectively explains 84% of the observed viral occurrence. Specifically, *Ae. albopictus* suitability (72.47%) and *Ae. aegypti* suitability (11.92%) emerged as the dominant predictors (**Table 1**). Beyond these biotic factors, the mean temperature of wettest quarter (bio8) contributed 9.89%. Response curves showed a threshold effect: when vector suitability was below threshold values (*Ae. aegypti* < 659, *Ae. albopictus* < 459), CHIKV occurrence probability plateaued at a low level. Above these thresholds, probability increased steadily with vector suitability. The response curve of bio8 showed a suitable range of 11.89 °C–36.91 °C, with a peak at 27.53 °C (**Supplementary Figure 6**).

To further quantify the predictive gain of incorporating biological factors and validate the necessity of the hierarchical framework, we conducted an ablation study comparing it to a climate-only baseline model. While the baseline model achieved comparable statistical accuracy (AUC = 0.903, TSS = 0.664; **Supplementary Table 6**), its driver structure shifted significantly. In the absence of vector suitability, the model was forced to incorporate variables previously excluded due to high multicollinearity with the predicted vector suitability layers (VIF > 10), such as bio3 and bio9, with their contributions surging to 31.00% and 24.24% respectively (**Supplementary Table 7**).

The prediction distributions of the four best-performing algorithms (GBM, MARS, MaxEnt, RF) are shown in **Figure 4**, and the results of the other six models are presented in **Supplementary Figure 7** (SRE was excluded). All models consistently highlighted high-risk areas in tropical and subtropical regions. The RF model produced the most conservative predictions, while GBM, MARS, and MaxEnt showed broader continuous risk areas, particularly in the Amazon rainforest, the Congo Basin, and northern Australia.

**Figure 7:**
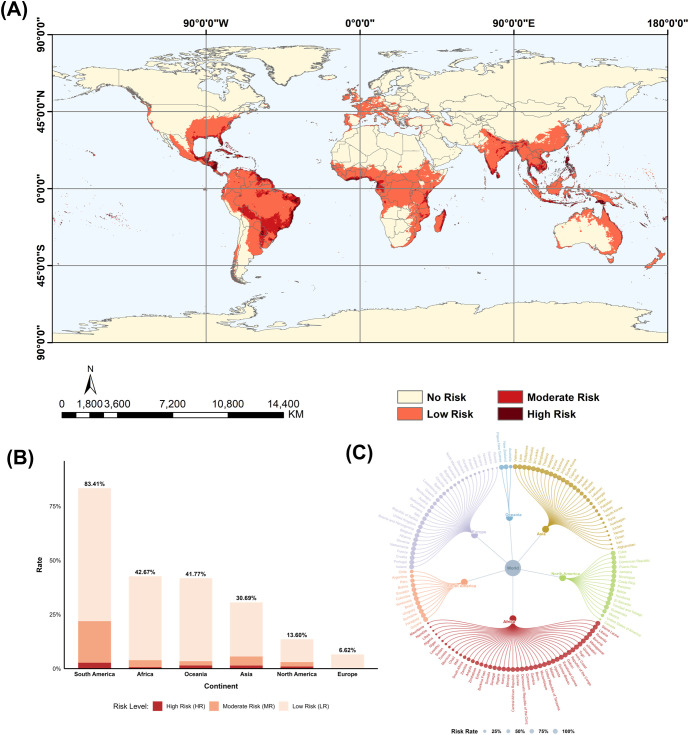
Ensemble model (EMwmean) projections for CHIKV. **(A)** Global potential distribution for CHIKV under current climatic conditions. **(B)** The predicted risk area percentage of CHIKV for each continent. **(C)** Risk rate across affected countries and regions. Circle size represents the level of total risk rates (the sum of low-risk rates, moderate-risk rates and high-risk rates).

The EMwmean prediction (**Figure 7A**) indicated that approximately 21.26% of the global land area is identified as a potential transmission risk zone for CHIKV. High-risk areas show coastal aggregation, distributed along the Caribbean coast of North America, eastern South America, the Gulf of Guinea coast of Africa, and coastal areas of South and Southeast Asia. These predicted high-risk zones closely align with the historical endemic areas confirmed by a comprehensive independent systematic literature review (Bettis et al., 2022). Moderate and low-risk areas extend from core zones to inland areas and higher latitudes.

At the continental scale (**Figure 7B**), South America had the highest risk proportion (83.41%), followed by Africa (42.67%), Oceania (41.77%), Asia (30.69%), North America (13.60%), and Europe (6.62%). At the national scale, CHIKV transmission risk covers 139 countries and regions (**Figure 7C**). Countries with high-risk exposure include Equatorial Guinea, Ivory Coast, Ghana, Madagascar, and Liberia in Africa; the Philippines, Sri Lanka, Cambodia, Bangladesh, and Thailand in Asia; Jamaica, Nicaragua, Belize, Puerto Rico, and Cuba in North America; Guyana, Suriname, Paraguay, Brazil, and Ecuador in South America (complete rankings in **Supplementary Table 8**).

### 3.5 Future potential distribution of CHIKV

**Figure 8:**
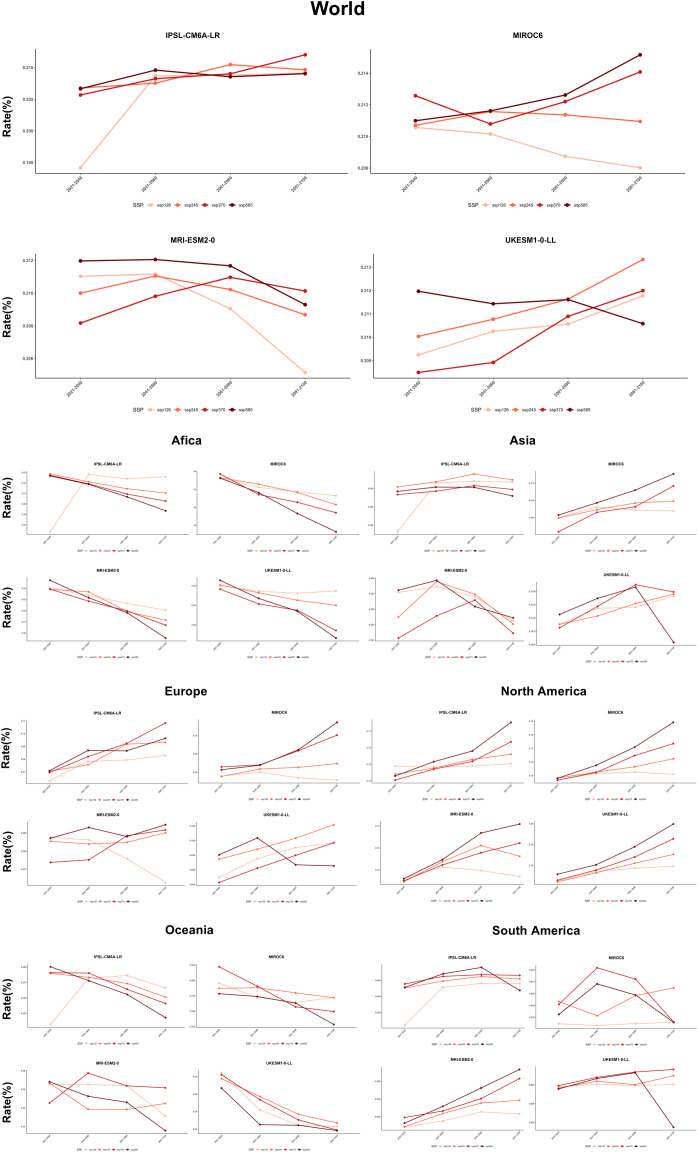
Variation in the total risk rates for the projected CHIKV risk areas globally and continentally under future climate scenarios. Colored lines represent four SSPs: SSP126 (light pink), SSP245 (orange), SSP370 (crimson), and SSP585 (dark brown). Results are discretized by GCM (panels) to explicitly illustrate inter-model structural uncertainty and reveal divergent risk trajectories.

Based on the CMIP6 framework, this study integrated four shared socioeconomic pathways (SSP126, SSP245, SSP370, and SSP585) and four global climate models (IPSL-CM6A-LR, MIROC6, MRI-ESM2-0, UKESM1-0-LL) to simulate CHIKV distribution risk across four future periods: 2021–2040, 2041–2060, 2061–2080, and 2081–2100. Changes in total risk rates globally and by continent are shown in **Figure 8**.

At the global scale, inter-model differences and inter-pathway heterogeneity were evident. In high-climate-sensitivity models (IPSL-CM6A-LR and UKESM1-0-LL), CHIKV risk showed an upward trend in 7 out of 8 SSP-GCM combinations (87.5%), with the exception of SSP585 in UKESM1-0-LL. In low-climate-sensitivity models (MRI-ESM2-0), most scenarios showed a downward trend. In moderate-climate-sensitivity models (MIROC6), risk decreased under SSP126 and SSP245 pathways but increased under SSP370 and SSP585.

At the continental scale, the distribution of potential risks for CHIKV exhibits significant regional heterogeneity. Overall, risk areas in Europe and North America are projected to expand, while those in Oceania and Africa are expected to contract.

**Figure 9:**
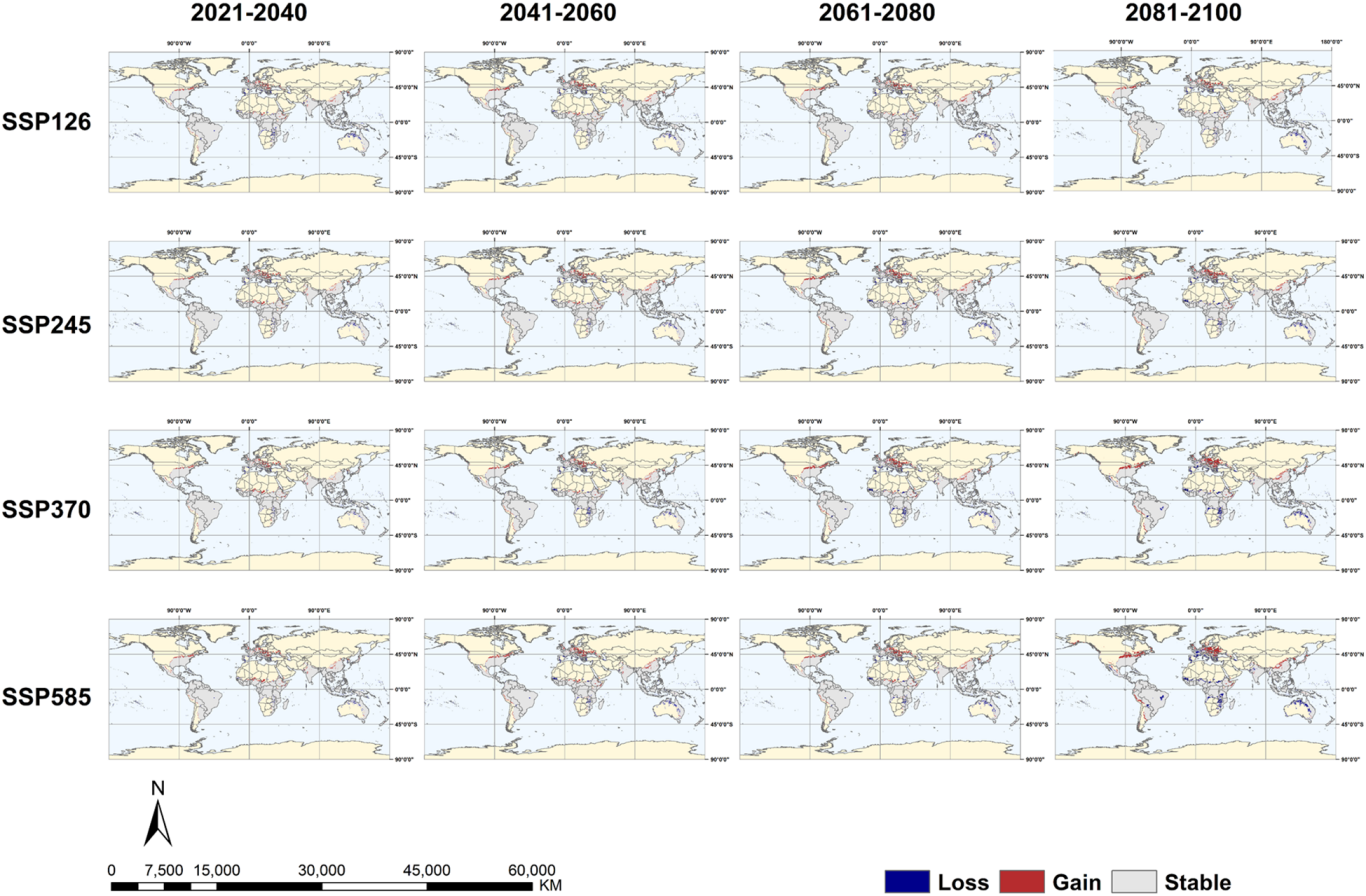
Changes in the potential distribution of CHIKV under future climate scenarios. This image illustrates the potential expansion and contraction of risk zones across future periods (2021–2040, 2041–2060, 2061–2080, 2081–2100) under four SSP scenarios (SSP126, SSP245, SSP370, SSP585). Red areas represent range expansion (Gain), blue areas represent range contraction (Loss), and gray areas indicate stable threatened areas (Stable).

To reveal spatial details of future CHIKV distribution, we integrated prediction data from all GCMs (**Figure 9**). Although the overall distribution range remains consistent with current patterns, both expansion and contraction trends are significant in marginal zones. Risk expansion hotspots include North America (northeastern United States and southeastern Canada), South America (Chile and Argentina), north-central Europe, and East Asia (China, Japan, and North Korea). Risk reduction areas are concentrated in the Mediterranean coast of Europe and northern Australia. The Sahel region in Africa exhibits pronounced temporal heterogeneity across the century: under SSP245, SSP370, and SSP585 pathways, this region shows expansion in the early period (2021–2040), but by 2081–2100, contraction becomes the dominant feature. To explicitly quantify and visualize this spatial uncertainty across the four GCMs, we calculated pixel-wise standard deviation (SD) maps (**Supplementary Figure 8**). These maps reveal that the highest inter-model uncertainty regarding future CHIKV distribution is predominantly concentrated in Europe and eastern North America, indicating that projections in these emerging high-risk zones exhibit the greatest variance among GCMs.

## 4 Discussion

In this study, a hierarchical modeling approach was used, which included the predicted habitat suitability of *Ae. aegypti* and *Ae. albopictus* as biological variables in the risk assessment of CHIKV (Xu et al., 2022). This design represents a key methodological innovation compared to previous approaches that treat climate, socio-economics, and vectors as parallel predictors. Our hierarchical framework first models vector niches, then uses vector suitability as mechanistic drivers of viral risk, offering advantages in biological realism, transferability, and scenario flexibility. Our finding that vectors explain 84% of CHIKV distribution validates this mechanistic approach.

The ablation study underscores the necessity of our hierarchical approach. While a climate-only baseline yielded comparable statistical metrics (AUC = 0.903, TSS = 0.664), its reliance on fragmented proxies (e.g., bio3 and bio9) highlights a structural flaw in conventional models. By explicitly integrating vector suitability, our framework replaces these indirect correlations with a more realistic, mechanistic causal chain. Thus, the primary advancement of this methodology lies not in incremental statistical gains, but in enhancing the biological realism and mechanistic depth of CHIKV risk projections.

Building on this mechanistic foundation, we employed a TSS-weighted ensemble approach (EMwmean) on the Biomod2 platform to further minimize methodological uncertainty. The final ensemble models exhibited excellent predictive accuracy (AUC = 0.909, TSS = 0.659). Although Random Forest (RF) carried the maximum weight due to its superior TSS scores, visual comparisons confirm it does not dominate the output. Instead, EMwmean effectively balances RF’s highly conservative spatial estimates with the broader projections of GBM and MaxEnt. This consensus mechanism reduces the risks of bias and ensures a robust, multi-algorithm result rather than a mere replica of a single high-weight model (Pacifici et al., 2019; Jia et al., 2023; Chen et al., 2025).

Our contemporary risk maps align well with recent CHIKV outbreak patterns. The 2014–2015 Americas epidemic (>2.9 million suspected and confirmed cases across 45 countries and territories) (Yactayo et al., 2016) occurred predominantly in regions we classified as risk areas. Similarly, the 2019–2020 resurgence in East Africa (Chinedu Eneh et al., 2023) matched our predicted risk zones in Kenya and Tanzania. Our projections indicate that 21.26% of global land area currently supports CHIKV transmission, encompassing 139 countries. This suggests that climate-driven vector expansion has significantly broadened the geographic scope of CHIKV threat.

As the primary vectors, *Ae. aegypti* and *Ae. albopictus* are ectothermic organisms whose reproduction and transmission cycles depend on warm and humid conditions (Reinhold et al., 2018; Xu et al., 2025b). Temperature modulates larval development and adult fecundity while regulating the pathogen’s EIP and infectivity (Reinhold et al., 2018; Robison et al., 2020). Our findings identify isothermality and temperature seasonality as critical predictors for *Ae. aegypti*, reflecting its requirement for thermal stability (Huber et al., 2018). This thermal sensitivity, especially the low egg hatching rate at 12 °C (Rojas Terrazas et al., 2020), imposes strict latitudinal limits on its distribution.

In contrast, *Ae. albopictus* exhibits broader ecological adaptation, as evidenced by its significantly wider bio3 (isothermality) range (29.02–71.79). This versatility is driven by its ability to produce cold-resistant diapausing eggs, enabling temperate strains to colonize high-latitude regions (Lounibos et al., 2003; Won and Choi, 2025). Nevertheless, bio9 (mean temperature of the driest quarter) remains a decisive constraint by serving as a proxy for winter conditions; previous research indicates that *Ae. albopictus* requires the mean temperature of the coldest quarter to exceed −3.21 °C for successful establishment (Xu et al., 2025a). Furthermore, precipitation (bio13) is another vital factor for its survival mechanisms (Germano et al., 2025; Martín et al., 2025). Given its superior ecological traits, *Ae. albopictus* (72.5%) plays a vastly more dominant role than *Ae. aegypti* (11.9%) in driving CHIKV distribution.

Beyond vector constraints, climate still played a direct regulatory role. Specifically, mean temperature of the wettest quarter (bio8) was the top bioclimatic predictor (9.89%). We propose bio8 primarily influences the extrinsic incubation period (EIP) rather than merely restricting vector ranges. Warmer temperatures (18–28 °C) shorten EIP and increase viral loads (Wimalasiri-Yapa et al., 2019), accelerating vector infectiousness (Robison et al., 2020) and elevating outbreak risks.

This study systematically evaluated the global transmission risk of CHIKV across diverse shared socioeconomic pathways (SSPs) and global climate models (GCMs). Previous research suggested a uniform downward trend in future CHIKV risk (Dai et al., 2025). In contrast, our projections reveal profound model heterogeneity and path dependency. Specifically, we clarify how the climate sensitivities of varying GCMs and the radiative forcing levels of different SSPs interact to drive the potential spread of CHIKV.

The contraction driven by extreme climate conditions is a primary manifestation of this heterogeneity. Specifically, in Africa, this reduction in CHIKV transmission risk is pervasive and occurs earlier. Under the SSP370 and SSP585 scenarios, all global climate models (GCMs) consistently predict a declining trend in risk prevalence from 2041 to 2100. Conversely, the contraction in Asia is delayed within more extreme scenarios. It is only under the SSP585 scenario during the late 21st century (2081–2100) that specific GCMs (IPSL-CM6A-LR, MRI-ESM2-0, and UKESM1-0-LL) project a contraction in transmission risk. This phenomenon is fundamentally associated with the non-linear response of vector suitability across tropical core regions. Our models identify isothermality (bio3) as a crucial predictor for vector distribution. The response curves demonstrate a sharp, non-linear decline in vector suitability when local climates fall outside specific stability windows. Although bio3 serves as a relative index of thermal stability rather than an absolute temperature metric, this statistical collapse aligns directly with mainstream physiological thermal thresholds (Liu et al., 2023; Covey et al., 2025). Established mechanistic models indicate that the absolute upper thermal limits for *Ae. aegypti* and *Ae. albopictus* are approximately 35 °C and 32 °C, respectively (Mordecai et al., 2019). Chronically exceeding these physiological ceilings leads to vector population collapse, thereby driving the projected contraction of CHIKV risk (Barr et al., 2024; Ryan et al., 2024).

This non-linear dynamic also mechanistically explains the high temporal heterogeneity observed in the Sahel region. As a transitional hydro-thermal ecotone, the Sahel exhibits a distinct ‘expansion-then-contraction’ trajectory driven by the vectors’ thermal performance curves. Initial warming (2021–2040) pushes the previously cooler sub-Saharan fringes into the optimal thermal window (Liu et al., 2023), thereby facilitating a transient spatial expansion of CHIKV risk. However, as extreme warming intensifies toward the late 21st century (2081–2100), temperatures in these newly colonized zones breach the vectors’ physiological limits. Consequently, the Sahel’s risk pattern represents a brief peak at the thermal optimum before contracting under lethal thermal stress and irregular precipitation.

In contrast to this tropical contraction, under the high-climate-sensitivity model (IPSL-CM6A-LR), global CHIKV risk exhibits a significant upward trend, consistent with the theory that climate warming alleviates low-temperature limitation in high-latitude regions (Liu-Helmersson et al., 2019). Our predictions reveal significant northward expansion into the northeastern United States, north-central Europe, and East Asia. However, as indicated by our spatial uncertainty analysis, projections for Europe and eastern North America also exhibit the highest inter-model standard deviation. This elevated uncertainty in temperate regions likely stems from their status as ecological margins for vector expansion. In these invasion fronts, even minor divergences among GCMs regarding future winter conditions can cross critical biological thresholds, notably the -10 °C (bio9) overwintering survival limit for *Ae. albopictus*, leading to starkly contrasting suitability projections among models.

Despite inter-model uncertainty, emerging hotspots at ecological invasion fronts are highly concerning. These densely populated, immunologically naive regions lack arboviral preparedness, risking large-scale epidemics reminiscent of the 2007 Italy outbreak (Rezza, 2018). To preempt such crises, countries along these temperate margins, including temperate Europe (e.g., the United Kingdom and Germany), the northeastern United States, and East Asia (e.g., China and Japan), must prioritize pre-emptive vector surveillance and clinical diagnosis training before 2040. Such infrastructure should include real-time genomic tracking of viruses and community-based mosquito control to mitigate the risk in these immunologically naive populations.

The critical interplay between climate forcing and such proactive societal adaptation is further revealed by the moderate-climate-sensitivity model (MIROC6). In this model, risk declines under SSP126 and SSP245, where moderate warming and embedded assumptions of sustainable development independently reduce transmission (Titcomb et al., 2024). Conversely, SSP370 and SSP585 combine aggressive warming with fragmented governance, creating ideal conditions for vector proliferation. Critically, even under high-emission scenarios, early policy interventions and infrastructure investments can decouple disease risk from climate trends (Zavaleta-Monestel et al., 2025), as demonstrated by Singapore’s success in controlling dengue through intensive vector surveillance and management (Ho et al., 2023).

The findings have several limitations. First, our occurrence records are subject to structural reporting biases, notably GBIF’s over-representation of Europe and North America and HealthMap’s potential under-reporting in Africa and Southeast Asia (Meyer et al., 2015). Although spatial thinning cannot entirely eliminate this macro-level bias, our hierarchical framework effectively buffers it. Second, while two-stage error propagation in the hierarchical approach is structurally mitigated via robust ensembling, future studies could employ Bayesian models to explicitly quantify this cascading uncertainty. Third, our models assume stationary vector-virus relationships, omitting unpredictable eco-evolutionary dynamics like viral mutations (Deeba et al., 2020). Thus, our projections isolate climate-driven niche expansions and likely represent a conservative baseline if future adaptations further enhance transmission efficiency. Finally, this study focused primarily on bioclimatic drivers, omitting crucial anthropogenic factors such as urbanization and population growth (Kraemer et al., 2019; Tozan et al., 2020). Future studies should integrate these multi-dimensional drivers to refine the predictive accuracy of infectious disease risk.

## 5 Conclusion

This study estimated CHIKV potential distribution under present and future conditions. The findings indicate that 139 countries face risk, covering 21.26% of global land area. Our models explained the primary role of vectors (84% contribution) and identified the direct influence of mean temperature of wettest quarter on viral transmission. While future projections show significant inter-model variability, North-central Europe, Northeastern North America, and Eastern Asia emerge as high-priority zones. We recommend that these regions implement proactive entomological surveillance and adaptive public health infrastructure by 2040 to mitigate the threat of emerging arboviral epidemics.

## Data Availability

Publicly available datasets were analyzed in this study. This data can be found here: Distribution data for *Ae. aegypti* and *Ae. albopictus* were obtained from the Global Biodiversity Information Facility (GBIF, https://www.gbif.org), and occurrence records for CHIKV were sourced from HealthMap (https://www.healthmap.org). Environmental variables were obtained from the WorldClim database (https://www.worldclim.org/). All databases were accessed in March 2025.

## Footnotes

1 https://www.gbif.org, accessed: March 2025.

2 https://www.healthmap.org, accessed: March 2025.

3 https://www.worldclim.org/, accessed: March 2025.
