# Supplementary material for: Predicting the global risk of chikungunya virus under climate change using ensemble species distribution models
Source: Front Cell Infect Microbiol. 2026 May 27;16:1808175. doi: 10.3389/fcimb.2026.1808175 (PMC13249597; doi:10.3389/fcimb.2026.1808175)
Supplement: Supplementary file 1 [file DataSheet1.docx]

Supplementary Material

# Supplementary Tables

**Supplementary Table 1. Information for variables.**

| Symbol | variables | spatial resolution | source |
| --- | --- | --- | --- |
| BIO1 | Annual Mean Temperature | 10 minutes | WorldClim (https://www.worldclim.org/) |
| BIO2 | Mean Diurnal Range (Mean of monthly (max temp - min temp)) | 10 minutes | WorldClim (https://www.worldclim.org/) |
| BIO3 | Isothermality (BIO2/BIO7) (×100) | 10 minutes | WorldClim (https://www.worldclim.org/) |
| BIO4 | Temperature Seasonality (standard deviation ×100) | 10 minutes | WorldClim (https://www.worldclim.org/) |
| BIO5 | Max Temperature of Warmest Month | 10 minutes | WorldClim (https://www.worldclim.org/) |
| BIO6 | Min Temperature of Coldest Month | 10 minutes | WorldClim (https://www.worldclim.org/) |
| BIO7 | Temperature Annual Range (BIO5-BIO6) | 10 minutes | WorldClim (https://www.worldclim.org/) |
| BIO8 | Mean Temperature of Wettest Quarter | 10 minutes | WorldClim (https://www.worldclim.org/) |
| BIO9 | Mean Temperature of Driest Quarter | 10 minutes | WorldClim (https://www.worldclim.org/) |
| BIO10 | Mean Temperature of Warmest Quarter | 10 minutes | WorldClim (https://www.worldclim.org/) |
| BIO11 | Mean Temperature of Coldest Quarter | 10 minutes | WorldClim (https://www.worldclim.org/) |
| BIO12 | Annual Precipitation | 10 minutes | WorldClim (https://www.worldclim.org/) |
| BIO13 | Precipitation of Wettest Month | 10 minutes | WorldClim (https://www.worldclim.org/) |
| BIO14 | Precipitation of Driest Month | 10 minutes | WorldClim (https://www.worldclim.org/) |
| BIO15 | Precipitation Seasonality (Coefficient of Variation) | 10 minutes | WorldClim (https://www.worldclim.org/) |
| BIO16 | Precipitation of Wettest Quarter | 10 minutes | WorldClim (https://www.worldclim.org/) |
| BIO17 | Precipitation of Driest Quarter | 10 minutes | WorldClim (https://www.worldclim.org/) |
| BIO18 | Precipitation of Warmest Quarter | 10 minutes | WorldClim (https://www.worldclim.org/) |
| BIO19 | Precipitation of Coldest Quarter | 10 minutes | WorldClim (https://www.worldclim.org/) |
| elevation | elevation | 10 minutes | WorldClim (https://www.worldclim.org/) |

**Supplementary Table 2. Model algorithms and parameter specifications.**

| Algorithm | Abbreviation | Key Hyperparameter Settings |
| --- | --- | --- |
| Artificial Neural Networks | ANN | Size: 2; Decay: default; Maxit: default |
| Classification Tree Analysis | CTA | Method: class; CP: 0.01 (default); Minbucket: default |
| Flexible Discriminant Analysis | FDA | Method: polyreg (Polynomial Regression) |
| Generalized Additive Models | GAM | Family: Binomial; Method: GCV.Cp; Smooth terms: s() |
| Generalized Boosted Models | GBM | n.trees: 100; interaction.depth: 1; shrinkage: 0.1; distribution: bernoulli |
| Generalized Linear Models | GLM | Formula: Quadratic (included x and x^2^ terms); Link: Logit |
| Multivariate Adaptive Regression Splines | MARS | Default settings (Degree: 1) |
| Maximum Entropy | MaxEnt | Regmult: 1; Add samples to background: TRUE |
| Random Forest | RF | mtry: 1; Type: Classification; ntree: 500 (implied default) |
| Surface Range Envelope | SRE | Quantile: 0.025 |
| Extreme Gradient Boosting | XGBoost | nrounds: 4; verbose: 1; objective: binary:logistic |

**Supplementary Table 3. Habitat suitability classification thresholds for CHIKV and its vectors.**

| Species | Threshold (Cutoff) | Median Threshold (*P_50_*) | High Threshold (*P_75_*) |
| --- | --- | --- | --- |
| CHIKV | 502 | 830 | 866 |
| *Aedes aegypti* | 693 | 869 | 890 |
| *Aedes albopictus* | 605 | 784 | 826 |

**Supplementary Table 4. Risk area ratio of *Ae. aegypti* in different countries.**

| **Continent** | **Country & Region** | **High Risk** | **Moderate Risk** | **Low Risk** | **Total Risk** |
| --- | --- | --- | --- | --- | --- |
| North America | Puerto Rico | 0.692 | 0.192 | 0.115 | 1.000 |
| North America | Jamaica | 0.000 | 0.378 | 0.622 | 1.000 |
| North America | Nicaragua | 0.003 | 0.048 | 0.949 | 1.000 |
| South America | Guyana | 0.000 | 0.006 | 0.994 | 1.000 |
| North America | Belize | 0.015 | 0.123 | 0.862 | 1.000 |
| North America | Honduras | 0.003 | 0.068 | 0.929 | 1.000 |
| North America | El Salvador | 0.032 | 0.274 | 0.694 | 1.000 |
| South America | Suriname | 0.000 | 0.000 | 1.000 | 1.000 |
| North America | Trinidad and Tobago | 0.000 | 0.000 | 1.000 | 1.000 |
| North America | Haiti | 0.170 | 0.557 | 0.273 | 1.000 |
| Africa | Ivory Coast | 0.000 | 0.000 | 0.998 | 0.998 |
| North America | Cuba | 0.000 | 0.167 | 0.830 | 0.997 |
| North America | Dominican Republic | 0.041 | 0.405 | 0.547 | 0.993 |
| Africa | Equatorial Guinea | 0.000 | 0.000 | 0.987 | 0.987 |
| Africa | Gabon | 0.000 | 0.000 | 0.983 | 0.983 |
| Africa | Liberia | 0.000 | 0.000 | 0.979 | 0.979 |
| Africa | Eswatini | 0.000 | 0.000 | 0.964 | 0.964 |
| South America | Brazil | 0.188 | 0.230 | 0.528 | 0.946 |
| North America | Guatemala | 0.000 | 0.033 | 0.909 | 0.942 |
| South America | Venezuela | 0.000 | 0.016 | 0.919 | 0.936 |
| North America | Panama | 0.000 | 0.000 | 0.929 | 0.929 |
| North America | Costa Rica | 0.000 | 0.000 | 0.919 | 0.919 |
| Asia | Sri Lanka | 0.000 | 0.000 | 0.908 | 0.908 |
| Africa | Madagascar | 0.082 | 0.095 | 0.719 | 0.895 |
| Africa | Republic of the Congo | 0.000 | 0.000 | 0.887 | 0.887 |
| Africa | Democratic Republic of the Congo | 0.000 | 0.000 | 0.850 | 0.850 |
| Africa | Togo | 0.000 | 0.000 | 0.808 | 0.808 |
| Africa | Burundi | 0.000 | 0.000 | 0.807 | 0.807 |
| Asia | Philippines | 0.000 | 0.000 | 0.792 | 0.792 |
| Africa | Central African Republic | 0.000 | 0.023 | 0.762 | 0.784 |
| Asia | Cambodia | 0.000 | 0.000 | 0.770 | 0.770 |
| Africa | Sierra Leone | 0.000 | 0.000 | 0.755 | 0.755 |
| Africa | Ghana | 0.000 | 0.000 | 0.750 | 0.750 |
| Africa | Uganda | 0.000 | 0.000 | 0.744 | 0.744 |
| Africa | Mozambique | 0.021 | 0.070 | 0.598 | 0.689 |
| Africa | Cameroon | 0.000 | 0.000 | 0.675 | 0.675 |
| South America | Bolivia | 0.044 | 0.085 | 0.526 | 0.655 |
| South America | Paraguay | 0.000 | 0.000 | 0.630 | 0.630 |
| Asia | Thailand | 0.000 | 0.000 | 0.623 | 0.623 |
| South America | Colombia | 0.000 | 0.000 | 0.621 | 0.621 |
| Africa | Benin | 0.000 | 0.000 | 0.543 | 0.543 |
| Oceania | Papua New Guinea | 0.000 | 0.001 | 0.535 | 0.536 |
| Africa | Kenya | 0.000 | 0.010 | 0.517 | 0.526 |
| Asia | Indonesia | 0.000 | 0.006 | 0.512 | 0.518 |
| Africa | Guinea | 0.000 | 0.000 | 0.517 | 0.517 |
| Africa | United Republic of Tanzania | 0.000 | 0.015 | 0.477 | 0.492 |
| South America | Peru | 0.022 | 0.020 | 0.411 | 0.452 |
| Africa | Ethiopia | 0.000 | 0.000 | 0.414 | 0.414 |
| Asia | Malaysia | 0.000 | 0.000 | 0.414 | 0.414 |
| South America | Ecuador | 0.003 | 0.043 | 0.326 | 0.372 |
| Africa | Nigeria | 0.000 | 0.000 | 0.370 | 0.370 |
| Asia | Vietnam | 0.000 | 0.000 | 0.331 | 0.331 |
| Asia | Bangladesh | 0.000 | 0.000 | 0.305 | 0.305 |
| North America | Mexico | 0.010 | 0.027 | 0.244 | 0.282 |
| Africa | Malawi | 0.000 | 0.003 | 0.277 | 0.280 |
| Africa | Somalia | 0.000 | 0.000 | 0.264 | 0.264 |
| Africa | Angola | 0.000 | 0.000 | 0.249 | 0.249 |
| Africa | Rwanda | 0.000 | 0.000 | 0.216 | 0.216 |
| Asia | Laos | 0.000 | 0.000 | 0.205 | 0.205 |
| Africa | Burkina Faso | 0.000 | 0.000 | 0.196 | 0.196 |
| Africa | Eritrea | 0.000 | 0.000 | 0.134 | 0.134 |
| Africa | South Africa | 0.000 | 0.000 | 0.115 | 0.115 |
| Europe | France | 0.000 | 0.000 | 0.101 | 0.101 |
| Africa | Zimbabwe | 0.000 | 0.000 | 0.083 | 0.083 |
| Asia | Myanmar | 0.000 | 0.000 | 0.071 | 0.071 |
| Asia | India | 0.000 | 0.000 | 0.048 | 0.048 |
| South America | Argentina | 0.000 | 0.000 | 0.038 | 0.038 |
| Oceania | Australia | 0.000 | 0.000 | 0.028 | 0.028 |
| Asia | Bhutan | 0.000 | 0.000 | 0.023 | 0.023 |
| Africa | Mali | 0.000 | 0.000 | 0.015 | 0.015 |
| Africa | Namibia | 0.000 | 0.000 | 0.010 | 0.010 |
| North America | United States of America | 0.000 | 0.000 | 0.008 | 0.008 |
| Asia | China | 0.000 | 0.000 | 0.001 | 0.001 |
| Oceania | New Zealand | 0.000 | 0.000 | 0.001 | 0.001 |
| Africa | Zambia | 0.000 | 0.000 | 0.001 | 0.001 |

**Supplementary Table 5. Risk area ratio of *Ae. albopictus* in different countries.**

| **Continent** | **Country & Region** | **High Risk** | **Moderate Risk** | **Low Risk** | **Total Risk** |
| --- | --- | --- | --- | --- | --- |
| North America | Cuba | 0.043 | 0.411 | 0.546 | 1.000 |
| North America | Haiti | 0.034 | 0.386 | 0.580 | 1.000 |
| North America | Puerto Rico | 0.231 | 0.231 | 0.538 | 1.000 |
| North America | Jamaica | 0.351 | 0.324 | 0.324 | 1.000 |
| North America | Nicaragua | 0.008 | 0.533 | 0.459 | 1.000 |
| Africa | Eswatini | 0.000 | 0.000 | 1.000 | 1.000 |
| Asia | Bangladesh | 0.018 | 0.328 | 0.654 | 1.000 |
| North America | Belize | 0.046 | 0.277 | 0.677 | 1.000 |
| North America | El Salvador | 0.000 | 0.000 | 1.000 | 1.000 |
| South America | Suriname | 0.000 | 0.000 | 1.000 | 1.000 |
| Africa | Equatorial Guinea | 0.104 | 0.442 | 0.455 | 1.000 |
| South America | Uruguay | 0.049 | 0.566 | 0.385 | 1.000 |
| North America | Trinidad and Tobago | 0.000 | 0.000 | 1.000 | 1.000 |
| Asia | Cambodia | 0.000 | 0.054 | 0.944 | 0.998 |
| Asia | Vietnam | 0.102 | 0.500 | 0.396 | 0.998 |
| Asia | Laos | 0.016 | 0.285 | 0.697 | 0.997 |
| South America | Guyana | 0.000 | 0.000 | 0.997 | 0.997 |
| Asia | Sri Lanka | 0.000 | 0.015 | 0.979 | 0.995 |
| North America | Honduras | 0.000 | 0.243 | 0.751 | 0.994 |
| Africa | Gabon | 0.013 | 0.146 | 0.821 | 0.980 |
| North America | Panama | 0.000 | 0.000 | 0.978 | 0.978 |
| Asia | Thailand | 0.000 | 0.018 | 0.955 | 0.974 |
| North America | Dominican Republic | 0.000 | 0.169 | 0.797 | 0.966 |
| Europe | Portugal | 0.000 | 0.000 | 0.963 | 0.963 |
| Africa | Ivory Coast | 0.001 | 0.061 | 0.886 | 0.948 |
| Europe | Croatia | 0.000 | 0.058 | 0.888 | 0.946 |
| Africa | Sierra Leone | 0.000 | 0.127 | 0.797 | 0.925 |
| North America | Costa Rica | 0.000 | 0.000 | 0.899 | 0.899 |
| Asia | Philippines | 0.009 | 0.252 | 0.617 | 0.878 |
| South America | Brazil | 0.082 | 0.138 | 0.657 | 0.878 |
| Europe | Ireland | 0.000 | 0.000 | 0.868 | 0.868 |
| North America | Guatemala | 0.018 | 0.170 | 0.660 | 0.848 |
| Europe | Albania | 0.000 | 0.009 | 0.817 | 0.826 |
| Asia | South Korea | 0.000 | 0.079 | 0.738 | 0.817 |
| Europe | Slovenia | 0.000 | 0.186 | 0.616 | 0.802 |
| Asia | Myanmar | 0.051 | 0.083 | 0.662 | 0.795 |
| Asia | Cyprus | 0.000 | 0.000 | 0.778 | 0.778 |
| Africa | Liberia | 0.000 | 0.093 | 0.676 | 0.769 |
| Africa | Togo | 0.000 | 0.000 | 0.762 | 0.762 |
| Europe | Bosnia and Herzegovina | 0.000 | 0.136 | 0.587 | 0.723 |
| Oceania | New Zealand | 0.000 | 0.001 | 0.721 | 0.722 |
| South America | Bolivia | 0.000 | 0.065 | 0.642 | 0.708 |
| Africa | Madagascar | 0.180 | 0.081 | 0.445 | 0.705 |
| Europe | France | 0.002 | 0.038 | 0.647 | 0.688 |
| Africa | Mozambique | 0.001 | 0.059 | 0.607 | 0.667 |
| South America | Paraguay | 0.252 | 0.144 | 0.237 | 0.633 |
| Africa | Central African Republic | 0.000 | 0.000 | 0.632 | 0.632 |
| Europe | Italy | 0.003 | 0.105 | 0.521 | 0.630 |
| Africa | Cameroon | 0.000 | 0.063 | 0.556 | 0.619 |
| Africa | Ghana | 0.000 | 0.027 | 0.568 | 0.595 |
| Asia | Nepal | 0.000 | 0.091 | 0.490 | 0.581 |
| Asia | Japan | 0.007 | 0.081 | 0.489 | 0.577 |
| South America | Venezuela | 0.000 | 0.000 | 0.558 | 0.558 |
| Africa | Malawi | 0.006 | 0.042 | 0.510 | 0.557 |
| Asia | Malaysia | 0.002 | 0.022 | 0.501 | 0.525 |
| Europe | Republic of Serbia | 0.000 | 0.022 | 0.467 | 0.489 |
| Europe | Greece | 0.000 | 0.000 | 0.488 | 0.488 |
| Africa | Republic of the Congo | 0.000 | 0.001 | 0.477 | 0.478 |
| South America | Colombia | 0.000 | 0.000 | 0.458 | 0.458 |
| Europe | Montenegro | 0.000 | 0.018 | 0.429 | 0.446 |
| Europe | Switzerland | 0.000 | 0.006 | 0.428 | 0.434 |
| Asia | Indonesia | 0.000 | 0.009 | 0.422 | 0.431 |
| Oceania | Papua New Guinea | 0.000 | 0.023 | 0.406 | 0.428 |
| Europe | Germany | 0.000 | 0.002 | 0.418 | 0.420 |
| Asia | India | 0.012 | 0.045 | 0.353 | 0.410 |
| Europe | Austria | 0.000 | 0.000 | 0.367 | 0.367 |
| Africa | Benin | 0.000 | 0.000 | 0.364 | 0.364 |
| South America | Argentina | 0.019 | 0.049 | 0.291 | 0.359 |
| Asia | Lebanon | 0.000 | 0.000 | 0.353 | 0.353 |
| Africa | Nigeria | 0.000 | 0.048 | 0.290 | 0.337 |
| Africa | United Republic of Tanzania | 0.001 | 0.039 | 0.288 | 0.329 |
| Europe | Netherlands | 0.000 | 0.000 | 0.299 | 0.299 |
| Asia | Bhutan | 0.068 | 0.045 | 0.174 | 0.288 |
| Asia | Georgia | 0.041 | 0.045 | 0.201 | 0.286 |
| Asia | Israel | 0.000 | 0.000 | 0.286 | 0.286 |
| South America | Ecuador | 0.000 | 0.000 | 0.281 | 0.281 |
| North America | United States of America | 0.082 | 0.068 | 0.110 | 0.261 |
| Europe | Spain | 0.000 | 0.001 | 0.256 | 0.257 |
| Europe | Czechia | 0.000 | 0.000 | 0.239 | 0.239 |
| Europe | United Kingdom | 0.000 | 0.000 | 0.238 | 0.238 |
| Europe | Belgium | 0.000 | 0.000 | 0.234 | 0.234 |
| North America | Mexico | 0.003 | 0.025 | 0.201 | 0.229 |
| Europe | Slovakia | 0.000 | 0.000 | 0.227 | 0.227 |
| Asia | China | 0.007 | 0.042 | 0.175 | 0.223 |
| South America | Chile | 0.000 | 0.000 | 0.217 | 0.217 |
| Africa | Democratic Republic of the Congo | 0.000 | 0.000 | 0.184 | 0.184 |
| Asia | Turkey | 0.000 | 0.002 | 0.177 | 0.179 |
| Africa | Guinea | 0.000 | 0.000 | 0.171 | 0.171 |
| Africa | Kenya | 0.000 | 0.003 | 0.166 | 0.169 |
| Asia | Brunei | 0.000 | 0.000 | 0.167 | 0.167 |
| Europe | Romania | 0.000 | 0.003 | 0.143 | 0.146 |
| South America | Peru | 0.000 | 0.009 | 0.126 | 0.136 |
| Europe | Bulgaria | 0.000 | 0.000 | 0.123 | 0.123 |
| Oceania | Australia | 0.005 | 0.012 | 0.098 | 0.115 |
| Africa | Burkina Faso | 0.000 | 0.000 | 0.115 | 0.115 |
| Africa | South Africa | 0.000 | 0.010 | 0.101 | 0.112 |
| Asia | Pakistan | 0.000 | 0.008 | 0.097 | 0.106 |
| Africa | Angola | 0.000 | 0.002 | 0.099 | 0.101 |
| Africa | Burundi | 0.000 | 0.000 | 0.096 | 0.096 |
| Africa | Uganda | 0.000 | 0.000 | 0.088 | 0.088 |
| Europe | North Macedonia | 0.000 | 0.000 | 0.071 | 0.071 |
| Europe | Poland | 0.000 | 0.000 | 0.066 | 0.066 |
| Africa | Tunisia | 0.000 | 0.000 | 0.049 | 0.049 |
| Africa | Zimbabwe | 0.000 | 0.003 | 0.040 | 0.043 |
| Africa | Guinea-Bissau | 0.000 | 0.000 | 0.042 | 0.042 |
| Europe | Hungary | 0.000 | 0.000 | 0.038 | 0.038 |
| Asia | Azerbaijan | 0.000 | 0.000 | 0.034 | 0.034 |
| Asia | Syria | 0.000 | 0.000 | 0.033 | 0.033 |
| Africa | Morocco | 0.000 | 0.000 | 0.033 | 0.033 |
| Asia | North Korea | 0.000 | 0.000 | 0.032 | 0.032 |
| Europe | Denmark | 0.000 | 0.000 | 0.030 | 0.030 |
| Africa | Somalia | 0.000 | 0.000 | 0.028 | 0.028 |
| Africa | Ethiopia | 0.000 | 0.000 | 0.019 | 0.019 |
| Europe | Ukraine | 0.000 | 0.003 | 0.015 | 0.018 |
| Africa | Algeria | 0.000 | 0.000 | 0.016 | 0.016 |
| Asia | Iran | 0.000 | 0.002 | 0.007 | 0.009 |
| Europe | Iceland | 0.000 | 0.000 | 0.009 | 0.009 |
| Asia | Oman | 0.000 | 0.000 | 0.007 | 0.007 |
| Europe | Norway | 0.000 | 0.000 | 0.005 | 0.005 |
| Europe | Sweden | 0.000 | 0.000 | 0.005 | 0.005 |
| Africa | Mali | 0.000 | 0.000 | 0.003 | 0.003 |
| North America | Canada | 0.000 | 0.000 | 0.003 | 0.003 |
| Europe | Russia | 0.000 | 0.000 | 0.001 | 0.001 |
| Africa | Libya | 0.000 | 0.000 | 0.001 | 0.001 |
| Africa | Zambia | 0.000 | 0.000 | 0.000 | 0.000 |
| Asia | Kazakhstan | 0.000 | 0.000 | 0.000 | 0.000 |

**Supplementary Table 6.** **Model performance metrics of CHIKV under different modeling frameworks.**

|  | AUC | | | TSS | | | |  |
| --- | --- | --- | --- | --- | --- | --- | --- | --- |
|  | baseline model | | hierarchical model | | baseline model | | hierarchical model | |
| ANN | 0.785±0.068* | 0.786±0.075* | | 0.555±0.128* | | 0.563±0.146* | |  |
| CTA | 0.835±0.015 | 0.819±0.019 | | 0.635±0.014 | | 0.616±0.019 | |  |
| FDA | 0.870±0.001 | 0.892±0.002 | | 0.626±0.008 | | 0.637±0.002 | |  |
| GAM | 0.873±0.002 | 0.893±0.002 | | 0.629±0.006 | | 0.638±0.004 | |  |
| GBM | 0.889±0.003 | 0.894±0.003 | | 0.652±0.006 | | 0.643±0.006 | |  |
| GLM | 0.881±0.002 | 0.894±0.003 | | 0.615±0.009 | | 0.651±0.006 | |  |
| MARS | 0.886±0.004 | 0.901±0.005 | | 0.647±0.010 | | 0.666±0.015 | |  |
| MaxEnt | 0.894±0.002 | 0.905±0.002 | | 0.653±0.005 | | 0.658±0.006 | |  |
| RF | 0.919±0.004 | 0.919±0.003 | | 0.703±0.010 | | 0.702±0.010 | |  |
| SRE | 0.500±0.000* | 0.500±0.000* | | 0.000±0.000* | | 0.000±0.000* | |  |
| XGBoost | 0.891±0.009 | 0.892±0.007 | | 0.674±0.011 | | 0.665±0.015 | |  |
| EMwmean | 0.903 | 0.909 | | 0.664 | | 0.659 | |  |

Values marked with an asterisk (*) indicate that the algorithm failed to meet the predefined inclusion thresholds (AUC > 0.8 and TSS > 0.6 for the CHIKV model). Algorithms with any metric marked by an asterisk for a given species were explicitly excluded from the final TSS-weighted mean ensemble model (EMwmean).

**Supplementary Table 7.** **Variable contribution of CHIKV under different modeling frameworks.**

| Variable | Symbol | Percent contribution of CHIKV | | |
| --- | --- | --- | --- | --- |
|  |  | baseline model | hierarchical model |  |
| Mean diurnal range | bio2 | 4.47 | 0.31 |  |
| Isothermality | bio3 | 31.00 | — |  |
| Temperature seasonality | bio4 | 0.80 | 1.89 |  |
| Mean temperature of wettest quarter | bio8 | 10.44 | 9.89 |  |
| Mean temperature of driest quarter | bio9 | 24.24 | — |  |
| Precipitation of wettest month | bio13 | 23.34 | 0.23 |  |
| Precipitation of driest Month | bio14 | 1.76 | 0.40 |  |
| Precipitation seasonality | bio15 | 0.86 | 1.48 |  |
| Precipitation of warmest quarter | bio18 | 0.56 | 0.16 |  |
| Precipitation of coldest quarter | bio19 | 1.88 | 0.50 |  |
| Elevation | Elevation | 0.65 | 0.75 |  |
| *Ae. albopictus* | *Ae. albopictus* | — | 72.47 |  |
| *Ae. aegypti* | *Ae. aegypti* | — | 11.92 |  |

**Supplementary Table 8. Risk area ratio of CHIKV in different countries.**

| **Continent** | **Country & Region** | **High Risk** | **Moderate Risk** | **Low Risk** | **Total Risk** |
| --- | --- | --- | --- | --- | --- |
| Asia | Vietnam | 0.065 | 0.385 | 0.550 | 1.000 |
| North America | Cuba | 0.480 | 0.517 | 0.003 | 1.000 |
| Asia | Laos | 0.024 | 0.229 | 0.746 | 1.000 |
| North America | Haiti | 0.273 | 0.523 | 0.205 | 1.000 |
| North America | Dominican Republic | 0.284 | 0.534 | 0.182 | 1.000 |
| Asia | Philippines | 0.519 | 0.220 | 0.261 | 1.000 |
| North America | Puerto Rico | 0.538 | 0.308 | 0.154 | 1.000 |
| North America | Jamaica | 0.946 | 0.054 | 0.000 | 1.000 |
| North America | Nicaragua | 0.880 | 0.120 | 0.000 | 1.000 |
| Asia | Cambodia | 0.142 | 0.675 | 0.183 | 1.000 |
| North America | Costa Rica | 0.115 | 0.405 | 0.480 | 1.000 |
| Africa | Sierra Leone | 0.000 | 0.028 | 0.972 | 1.000 |
| Asia | Sri Lanka | 0.313 | 0.595 | 0.092 | 1.000 |
| North America | Panama | 0.018 | 0.489 | 0.493 | 1.000 |
| South America | Guyana | 0.259 | 0.473 | 0.268 | 1.000 |
| Africa | Liberia | 0.082 | 0.075 | 0.843 | 1.000 |
| Africa | Rwanda | 0.000 | 0.000 | 1.000 | 1.000 |
| Africa | Burundi | 0.000 | 0.048 | 0.952 | 1.000 |
| Africa | Madagascar | 0.090 | 0.219 | 0.691 | 1.000 |
| South America | Paraguay | 0.111 | 0.326 | 0.563 | 1.000 |
| Africa | Eswatini | 0.000 | 0.000 | 1.000 | 1.000 |
| Europe | Ireland | 0.000 | 0.000 | 1.000 | 1.000 |
| Asia | Bangladesh | 0.130 | 0.558 | 0.312 | 1.000 |
| Asia | Thailand | 0.090 | 0.203 | 0.707 | 1.000 |
| North America | Belize | 0.631 | 0.354 | 0.015 | 1.000 |
| North America | Honduras | 0.261 | 0.629 | 0.110 | 1.000 |
| North America | El Salvador | 0.355 | 0.565 | 0.081 | 1.000 |
| Africa | Ghana | 0.124 | 0.216 | 0.660 | 1.000 |
| Africa | Togo | 0.006 | 0.180 | 0.814 | 1.000 |
| Africa | Ivory Coast | 0.137 | 0.130 | 0.734 | 1.000 |
| Asia | Malaysia | 0.030 | 0.160 | 0.809 | 1.000 |
| South America | Suriname | 0.193 | 0.329 | 0.478 | 1.000 |
| Africa | Republic of the Congo | 0.000 | 0.012 | 0.988 | 1.000 |
| Africa | Gabon | 0.007 | 0.545 | 0.448 | 1.000 |
| Africa | Equatorial Guinea | 0.481 | 0.519 | 0.000 | 1.000 |
| South America | Uruguay | 0.000 | 0.356 | 0.644 | 1.000 |
| Africa | Guinea-Bissau | 0.000 | 0.042 | 0.958 | 1.000 |
| North America | Trinidad and Tobago | 0.333 | 0.667 | 0.000 | 1.000 |
| Asia | Brunei | 0.000 | 0.000 | 1.000 | 1.000 |
| Africa | Uganda | 0.000 | 0.020 | 0.980 | 1.000 |
| North America | Guatemala | 0.280 | 0.410 | 0.310 | 1.000 |
| South America | Brazil | 0.041 | 0.270 | 0.688 | 1.000 |
| South America | Venezuela | 0.004 | 0.144 | 0.851 | 1.000 |
| Europe | Portugal | 0.000 | 0.000 | 0.997 | 0.997 |
| Africa | Malawi | 0.000 | 0.196 | 0.798 | 0.994 |
| Asia | Myanmar | 0.033 | 0.162 | 0.799 | 0.993 |
| Oceania | Papua New Guinea | 0.142 | 0.075 | 0.776 | 0.993 |
| Asia | Indonesia | 0.073 | 0.117 | 0.803 | 0.993 |
| Africa | United Republic of Tanzania | 0.030 | 0.114 | 0.846 | 0.990 |
| South America | Colombia | 0.000 | 0.018 | 0.972 | 0.990 |
| Africa | Mozambique | 0.000 | 0.176 | 0.811 | 0.988 |
| Africa | Benin | 0.003 | 0.067 | 0.903 | 0.974 |
| Europe | Croatia | 0.000 | 0.000 | 0.973 | 0.973 |
| Africa | Gambia | 0.000 | 0.000 | 0.971 | 0.971 |
| Africa | Cameroon | 0.038 | 0.195 | 0.736 | 0.969 |
| Africa | Democratic Republic of the Congo | 0.000 | 0.001 | 0.959 | 0.960 |
| Oceania | New Zealand | 0.000 | 0.001 | 0.955 | 0.956 |
| Africa | Guinea | 0.000 | 0.023 | 0.917 | 0.941 |
| South America | Ecuador | 0.016 | 0.149 | 0.763 | 0.928 |
| Europe | France | 0.028 | 0.033 | 0.861 | 0.922 |
| Africa | Central African Republic | 0.000 | 0.000 | 0.903 | 0.903 |
| Europe | Netherlands | 0.000 | 0.000 | 0.898 | 0.898 |
| Asia | South Korea | 0.000 | 0.000 | 0.896 | 0.896 |
| Europe | Slovenia | 0.000 | 0.000 | 0.895 | 0.895 |
| Europe | Albania | 0.000 | 0.000 | 0.890 | 0.890 |
| Asia | Cyprus | 0.000 | 0.000 | 0.889 | 0.889 |
| Africa | Ethiopia | 0.000 | 0.000 | 0.880 | 0.880 |
| Asia | India | 0.019 | 0.145 | 0.713 | 0.877 |
| Africa | Kenya | 0.015 | 0.107 | 0.716 | 0.838 |
| Europe | Belgium | 0.000 | 0.000 | 0.810 | 0.810 |
| South America | Bolivia | 0.011 | 0.444 | 0.333 | 0.788 |
| Africa | Nigeria | 0.003 | 0.094 | 0.677 | 0.775 |
| Europe | Bosnia and Herzegovina | 0.000 | 0.000 | 0.775 | 0.775 |
| Asia | Nepal | 0.000 | 0.132 | 0.622 | 0.754 |
| Europe | United Kingdom | 0.000 | 0.000 | 0.728 | 0.728 |
| Africa | Senegal | 0.000 | 0.000 | 0.698 | 0.698 |
| South America | Peru | 0.000 | 0.049 | 0.643 | 0.692 |
| Europe | Republic of Serbia | 0.000 | 0.000 | 0.688 | 0.688 |
| Europe | Italy | 0.000 | 0.000 | 0.662 | 0.662 |
| Africa | Somalia | 0.000 | 0.030 | 0.628 | 0.658 |
| North America | Mexico | 0.029 | 0.079 | 0.537 | 0.646 |
| Asia | Japan | 0.000 | 0.000 | 0.637 | 0.637 |
| Europe | Germany | 0.000 | 0.000 | 0.596 | 0.596 |
| Europe | Switzerland | 0.000 | 0.000 | 0.561 | 0.561 |
| Africa | Burkina Faso | 0.000 | 0.000 | 0.556 | 0.556 |
| Africa | Eritrea | 0.000 | 0.000 | 0.544 | 0.544 |
| Asia | Bhutan | 0.000 | 0.076 | 0.455 | 0.530 |
| Africa | Zimbabwe | 0.000 | 0.004 | 0.520 | 0.525 |
| Europe | Austria | 0.000 | 0.000 | 0.487 | 0.487 |
| Europe | Montenegro | 0.000 | 0.000 | 0.482 | 0.482 |
| Africa | Angola | 0.000 | 0.015 | 0.455 | 0.470 |
| South America | Argentina | 0.001 | 0.043 | 0.412 | 0.456 |
| Europe | Greece | 0.000 | 0.000 | 0.455 | 0.455 |
| Asia | Israel | 0.000 | 0.000 | 0.429 | 0.429 |
| Europe | Luxembourg | 0.000 | 0.000 | 0.417 | 0.417 |
| Asia | Lebanon | 0.000 | 0.000 | 0.412 | 0.412 |
| Europe | Spain | 0.000 | 0.000 | 0.410 | 0.410 |
| South America | Chile | 0.000 | 0.000 | 0.391 | 0.391 |
| Africa | Zambia | 0.000 | 0.000 | 0.382 | 0.382 |
| Oceania | Australia | 0.003 | 0.015 | 0.338 | 0.356 |
| Africa | South Africa | 0.002 | 0.015 | 0.282 | 0.298 |
| North America | United States of America | 0.004 | 0.032 | 0.246 | 0.282 |
| Asia | Georgia | 0.000 | 0.000 | 0.257 | 0.257 |
| Asia | China | 0.000 | 0.003 | 0.233 | 0.236 |
| Asia | Pakistan | 0.000 | 0.004 | 0.229 | 0.233 |
| Europe | Czechia | 0.000 | 0.000 | 0.223 | 0.223 |
| Europe | Slovakia | 0.000 | 0.000 | 0.213 | 0.213 |
| Europe | Bulgaria | 0.000 | 0.000 | 0.202 | 0.202 |
| Europe | Romania | 0.000 | 0.000 | 0.168 | 0.168 |
| Asia | Turkey | 0.000 | 0.000 | 0.154 | 0.154 |
| Europe | North Macedonia | 0.000 | 0.000 | 0.143 | 0.143 |
| Asia | North Korea | 0.000 | 0.000 | 0.127 | 0.127 |
| Europe | Denmark | 0.000 | 0.000 | 0.107 | 0.107 |
| Africa | Mali | 0.000 | 0.000 | 0.105 | 0.105 |
| Africa | Chad | 0.000 | 0.000 | 0.085 | 0.085 |
| Africa | Morocco | 0.000 | 0.000 | 0.078 | 0.078 |
| Africa | Tunisia | 0.000 | 0.000 | 0.077 | 0.077 |
| Europe | Hungary | 0.000 | 0.000 | 0.071 | 0.071 |
| Europe | Poland | 0.000 | 0.000 | 0.060 | 0.060 |
| Africa | Sudan | 0.000 | 0.000 | 0.056 | 0.056 |
| Europe | Iceland | 0.000 | 0.000 | 0.047 | 0.047 |
| Asia | Syria | 0.000 | 0.000 | 0.040 | 0.040 |
| Africa | Lesotho | 0.000 | 0.000 | 0.039 | 0.039 |
| Asia | Azerbaijan | 0.000 | 0.000 | 0.027 | 0.027 |
| Africa | Niger | 0.000 | 0.000 | 0.018 | 0.018 |
| Europe | Ukraine | 0.000 | 0.000 | 0.016 | 0.016 |
| Asia | Jordan | 0.000 | 0.000 | 0.013 | 0.013 |
| Asia | Yemen | 0.000 | 0.000 | 0.013 | 0.013 |
| Africa | Algeria | 0.000 | 0.000 | 0.012 | 0.012 |
| Asia | Oman | 0.000 | 0.000 | 0.010 | 0.010 |
| Asia | Iran | 0.000 | 0.000 | 0.010 | 0.010 |
| Europe | Norway | 0.000 | 0.000 | 0.009 | 0.009 |
| Africa | Libya | 0.000 | 0.000 | 0.009 | 0.009 |
| North America | Canada | 0.000 | 0.000 | 0.007 | 0.007 |
| Asia | Afghanistan | 0.000 | 0.000 | 0.005 | 0.005 |
| Europe | Sweden | 0.000 | 0.000 | 0.004 | 0.004 |
| Africa | Namibia | 0.000 | 0.000 | 0.003 | 0.003 |
| Africa | Mauritania | 0.000 | 0.000 | 0.002 | 0.002 |
| Europe | Russia | 0.000 | 0.000 | 0.001 | 0.001 |

# Supplementary Figures

**
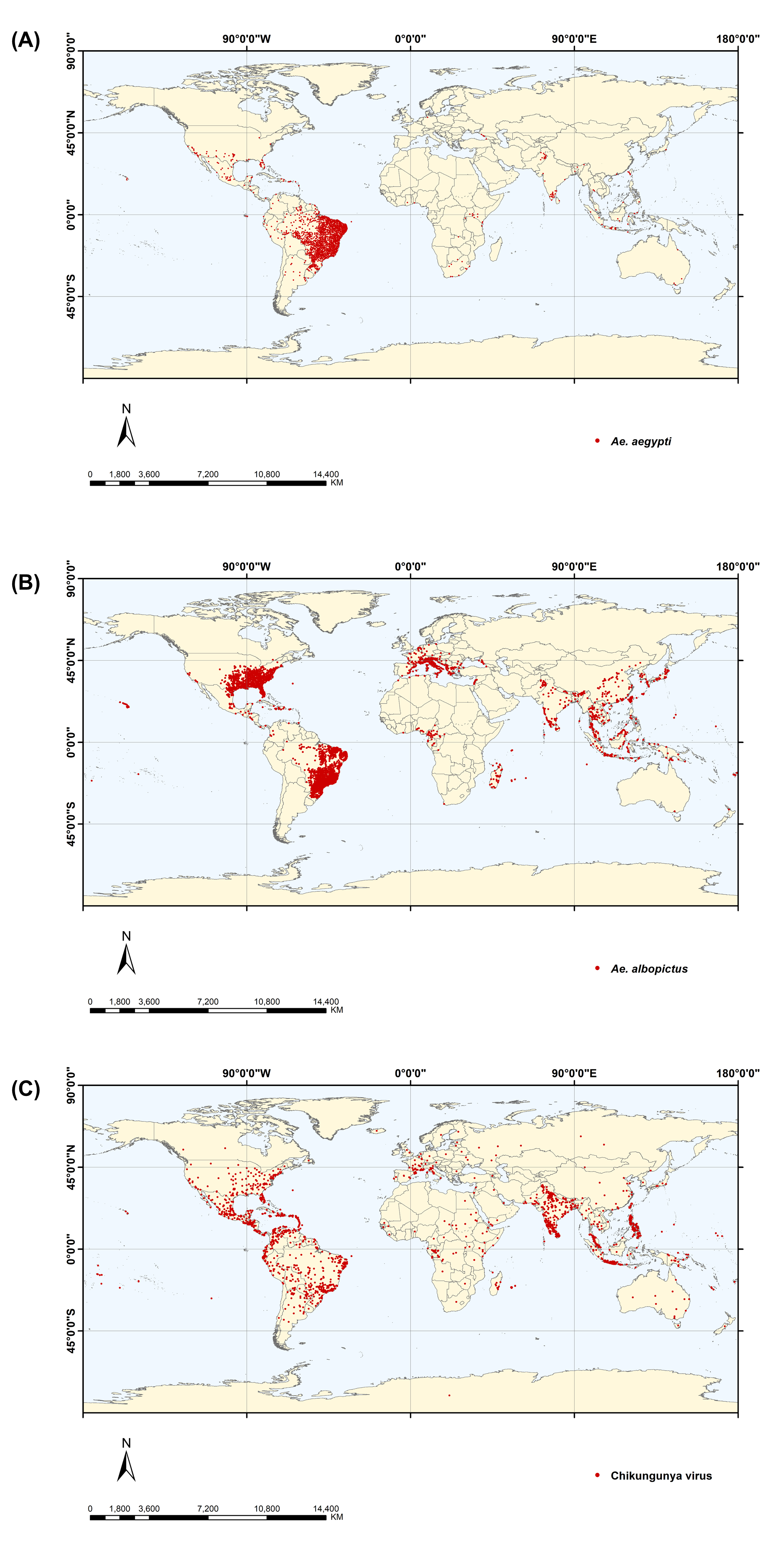
**

**Supplementary Figure 1.** Occurrence of *Ae. aegypti* (A), *Ae. albopictus* (B), Chikungunya virus (C).

**
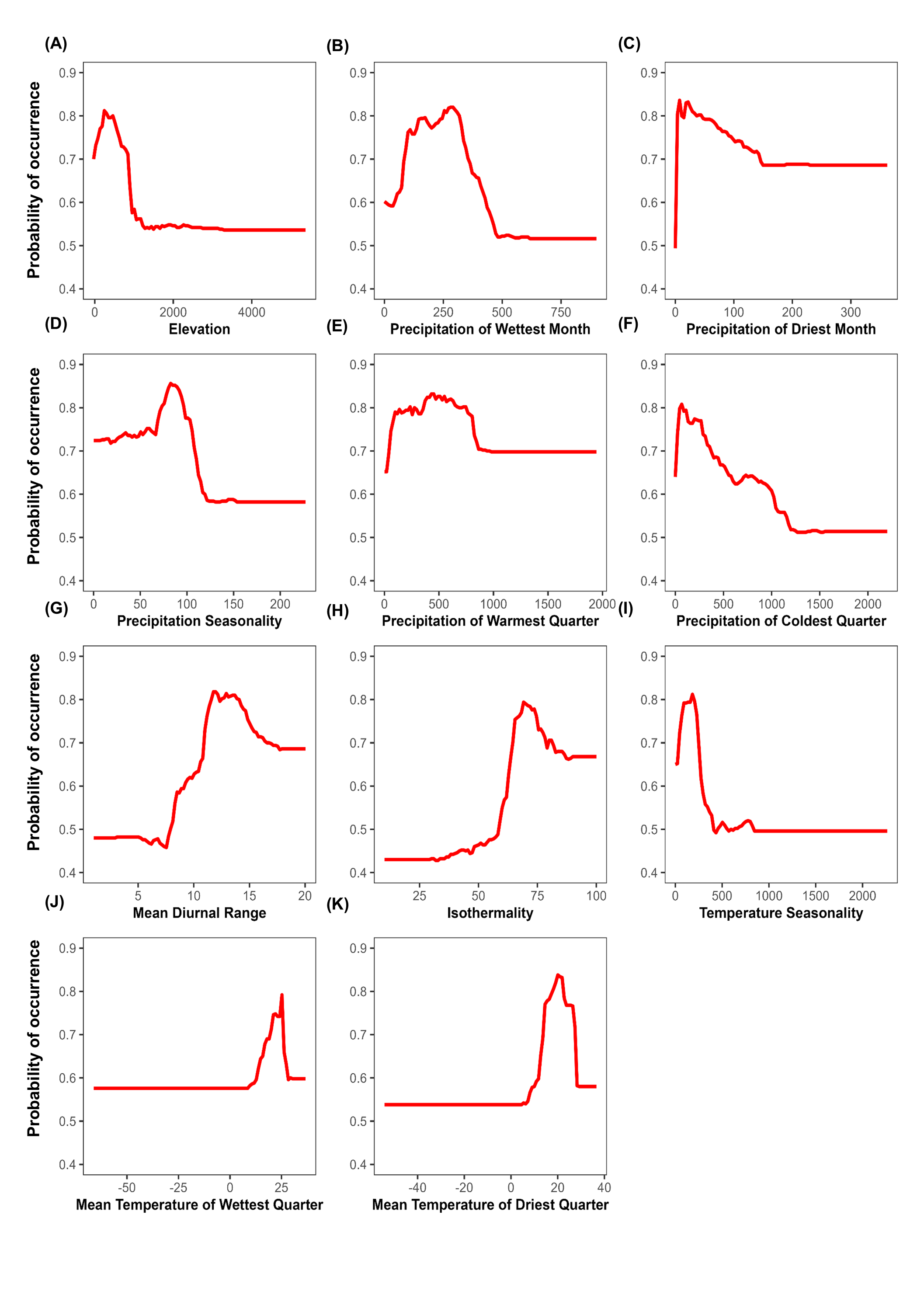
**

**Supplementary Figure 2.** Response curves between the distribution probability of *Ae. aegypti* and key variables*.*

**
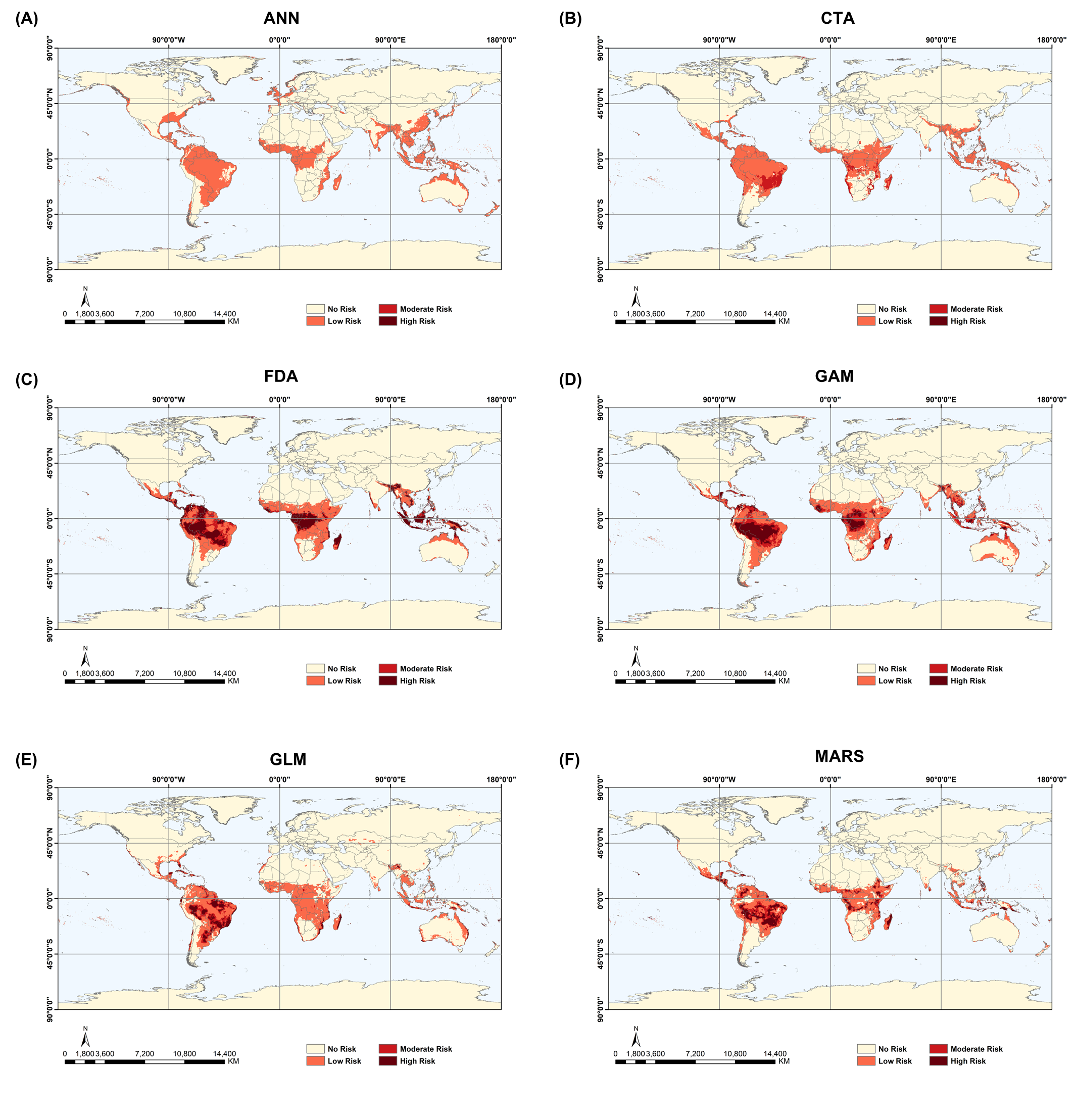
**

**Supplementary Figure 3.** Potential global distribution of *Ae. aegypti* under current climatic conditions, as predicted by six individual algorithms. (A) Artificial Neural Network (ANN), (B) Classification Tree Analysis (CTA), (C) Flexible Discriminant Analysis (FDA), (D) Generalized Additive Model (GAM), (E) Generalized Linear Model (GLM), and (F) Multivariate Adaptive Regression Splines (MARS).

**
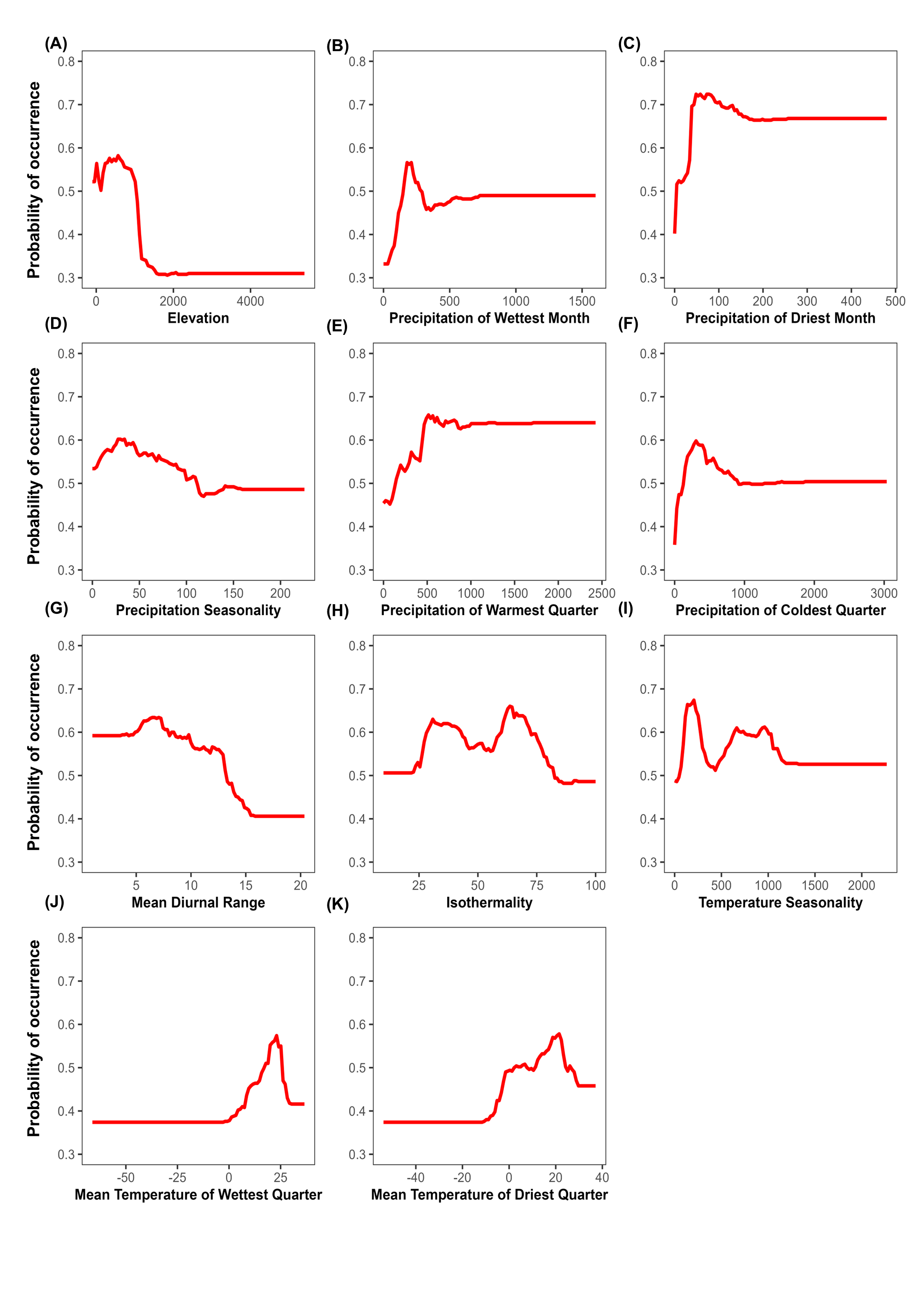
**

**Supplementary Figure 4.** Response curves between the distribution probability of *Ae. albopictus* and key variables*.*

**
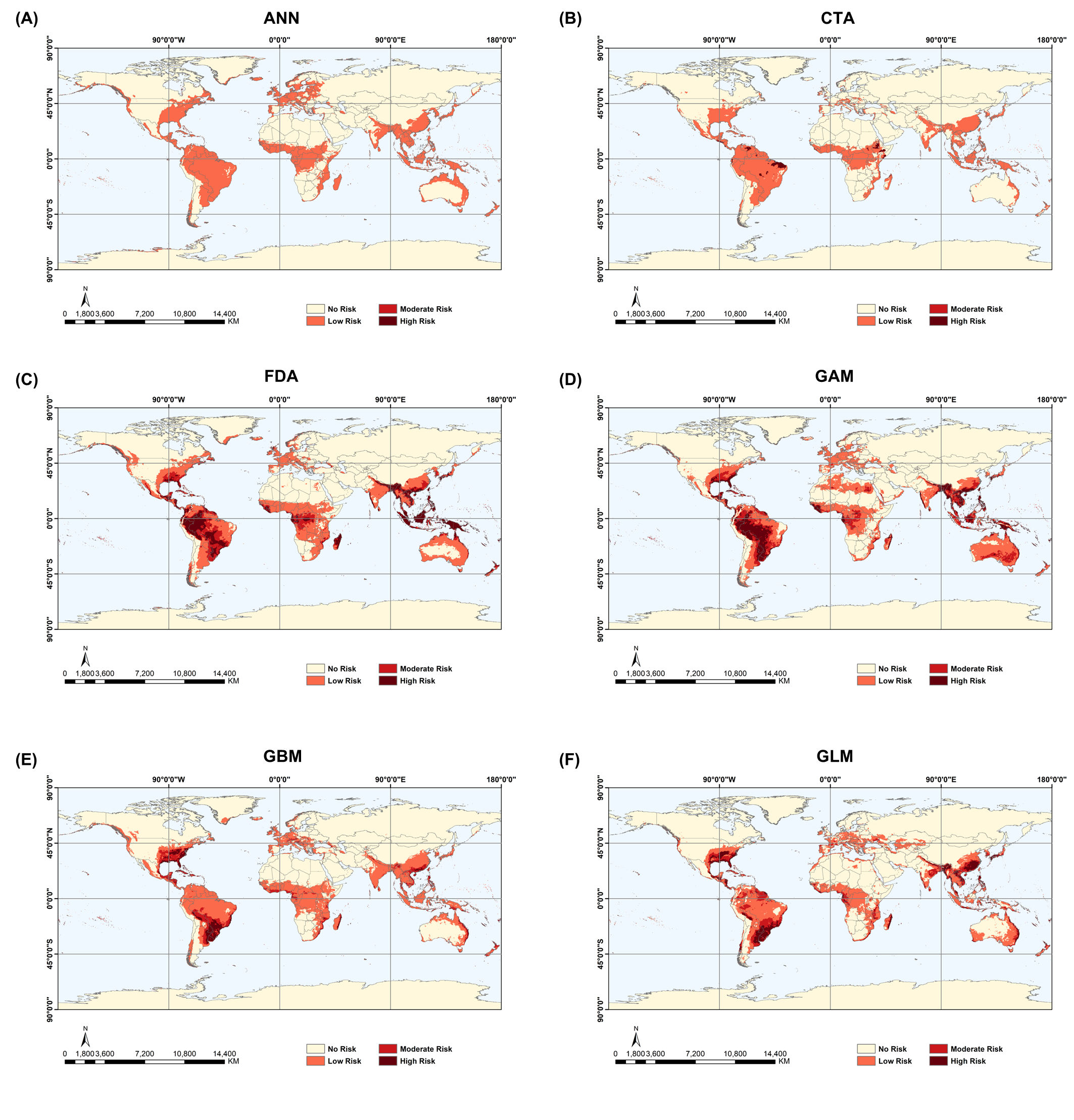
**

**Supplementary Figure 5.** Potential global distribution of *Ae. albopictus* under current climatic conditions, as predicted by six individual algorithms. (A) Artificial Neural Network (ANN), (B) Classification Tree Analysis (CTA), (C) Flexible Discriminant Analysis (FDA), (D) Generalized Additive Model (GAM), (E) Gradient Boosting Machine (GBM), and (F) Generalized Linear Model (GLM).


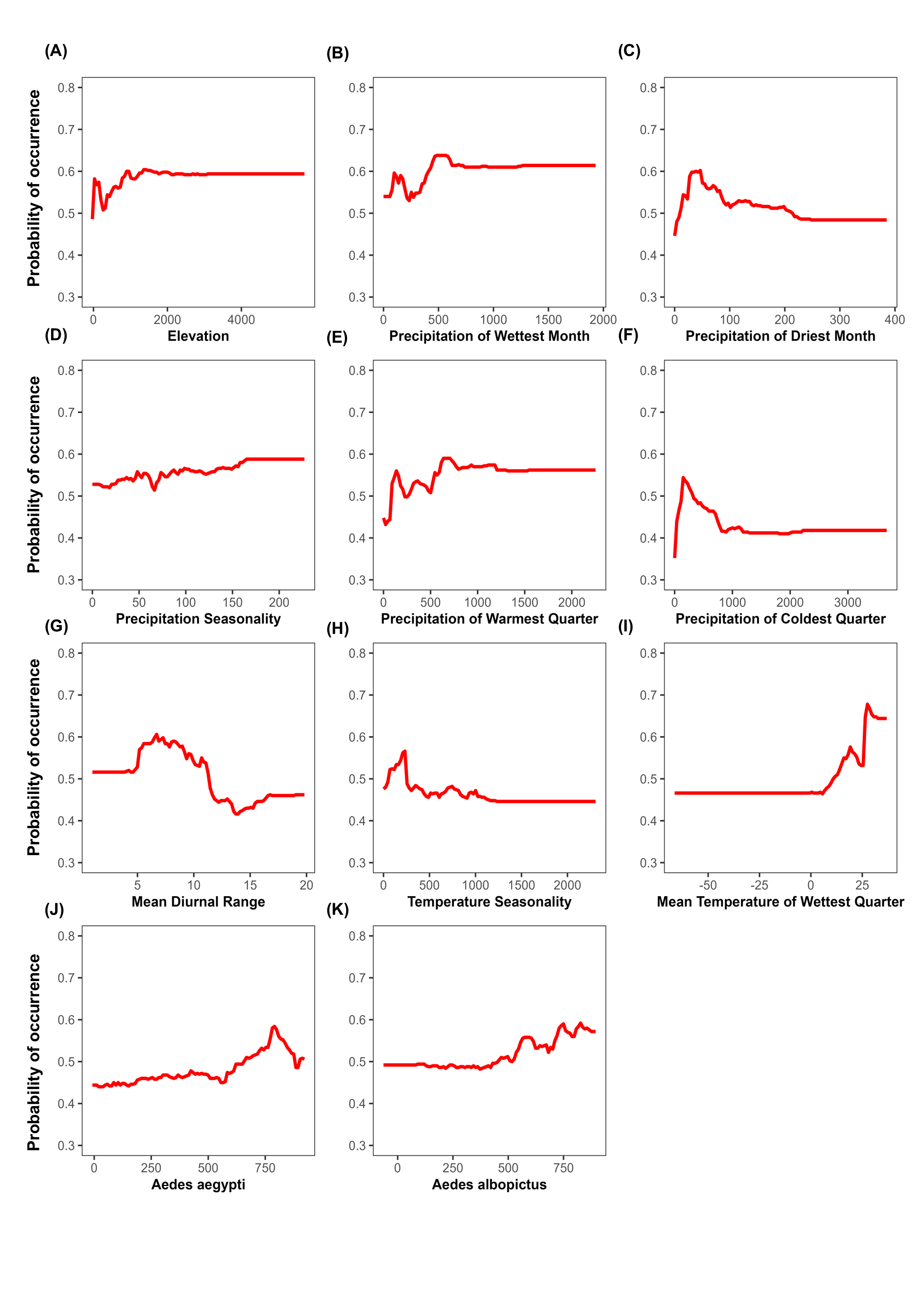


**Supplementary Figure 6.** Response curves between the distribution probability of CHIKV and key variables.

**
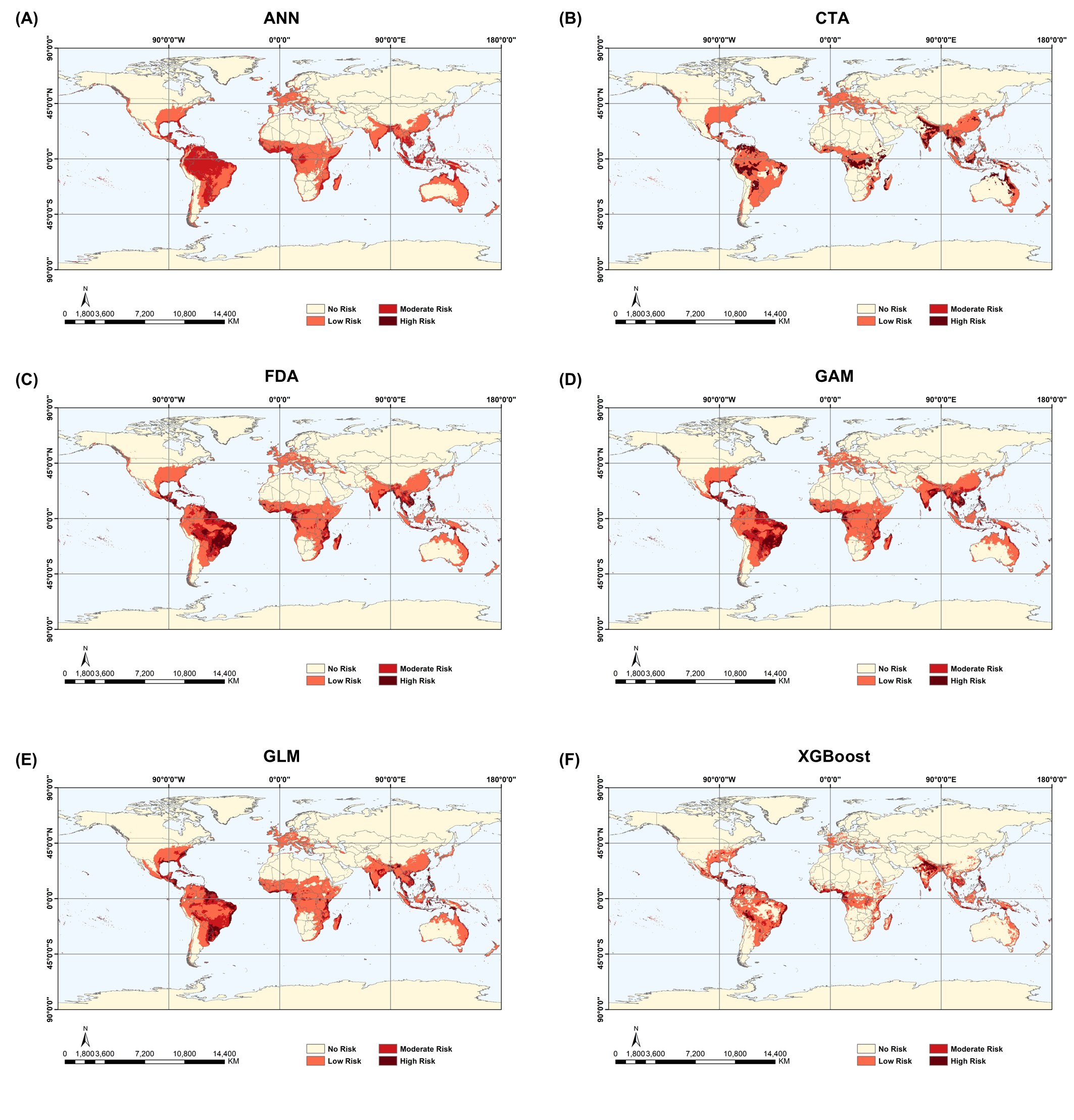
Supplementary Figure 7.** Potential global distribution of CHIKV under current climatic conditions, as predicted by six individual algorithms. (A) Artificial Neural Network (ANN), (B) Classification Tree Analysis (CTA), (C) Flexible Discriminant Analysis (FDA), (D) Generalized Additive Model (GAM), (E) Generalized Linear Model (GLM), and (F) Extreme Gradient Boosting (XGBoost).

**
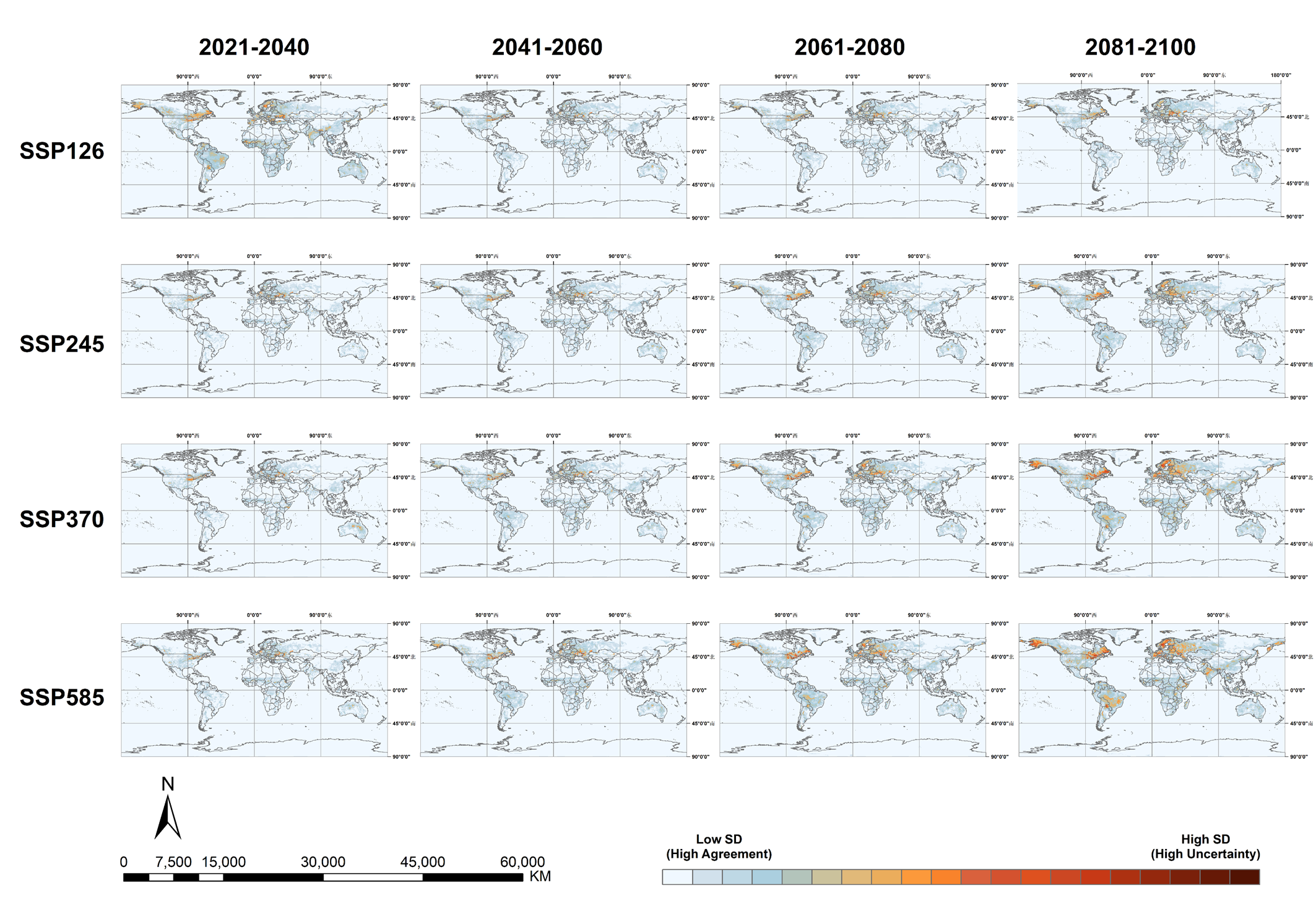
**

**Supplementary Figure 8.** Pixel-wise standard deviation (SD) maps of Chikungunya virus (CHIKV) distribution risk across four global climate models (GCMs). High SD values indicate high inter-model uncertainty, while low SD values indicate strong consensus among models.
